# Label-Free Refractive-Index-Based Detection of Breast, Leukemia, and Prostate Cancer Cells Using a Tetra-Core PCF SPR Biosensor

**DOI:** 10.3390/bios16090463

**Published:** 2026-08-25

**Authors:** Amit Kumar Shakya, Mantas Grigalavičius

**Affiliations:** Laser Research Center, Vilnius University, Saulėtekio al. 10, Vilnius, 10223 Vilniaus m. sav., Lithuania; mantas.grigalavicius@cr.vu.lt

**Keywords:** cancer, biosensor, refractive index, photonic crystal fiber, surface plasmon resonance, label-free detection

## Abstract

In this research, a high-performance plasmonic refractive index (RI) biosensor based on an external metal deposition (EMD) technique and photonic crystal fiber (PCF) platform for potential cancer detection is presented, investigated, and linked with real-time cancer cells. Variations in the RI of biological fluids are closely associated with pathological conditions, including cancer, due to changes in cellular composition and biomolecular concentration. The proposed tetra-core PCF–SPR biosensor operates within the biologically relevant RI range of 1.33–1.37, enabling the detection of subtle RI variations corresponding to various cancerous cells. The sensing mechanism of the proposed sensor is based on surface plasmon resonance (SPR) and analyzed using coupled mode light theory for both x- and y- polarized modes. Key sensing performance parameters, including confinement loss (CL), wavelength sensitivity (WS), amplitude sensitivity (AS), sensor resolution (SR), and figure of merit (FOM) are systematically evaluated. The biosensor reports a WS of 9769 and 9069 nm/RIU for x-pol. and y-pol., respectively, AS of 623.182 and $645.087\;{RIU}^{-1}$ for x-pol. and y-pol. respectively, SR in the order of $10^{-5}$ RIU, coefficient of determination ($R^{2}$) of 0.97 and 0.96, and FOM of 60.17 and $53.01\;{RIU}^{-1}$ for x-pol. and y-pol., respectively. Thus, the proposed PCF–SPR biosensor exhibits a dynamic range of 0.04 RIU. The sensing results demonstrate high sensitivity and strong resonance characteristics, indicating the capability of the proposed biosensor for label-free and non-invasive detection of cancer-associated RI changes in biological fluids. Thus, the presented biosensor offers a promising approach for the highly sensitive label-free detection of early-stage cancer cells by photonics sensing application.

## 1. Introduction

**Figure 1:**
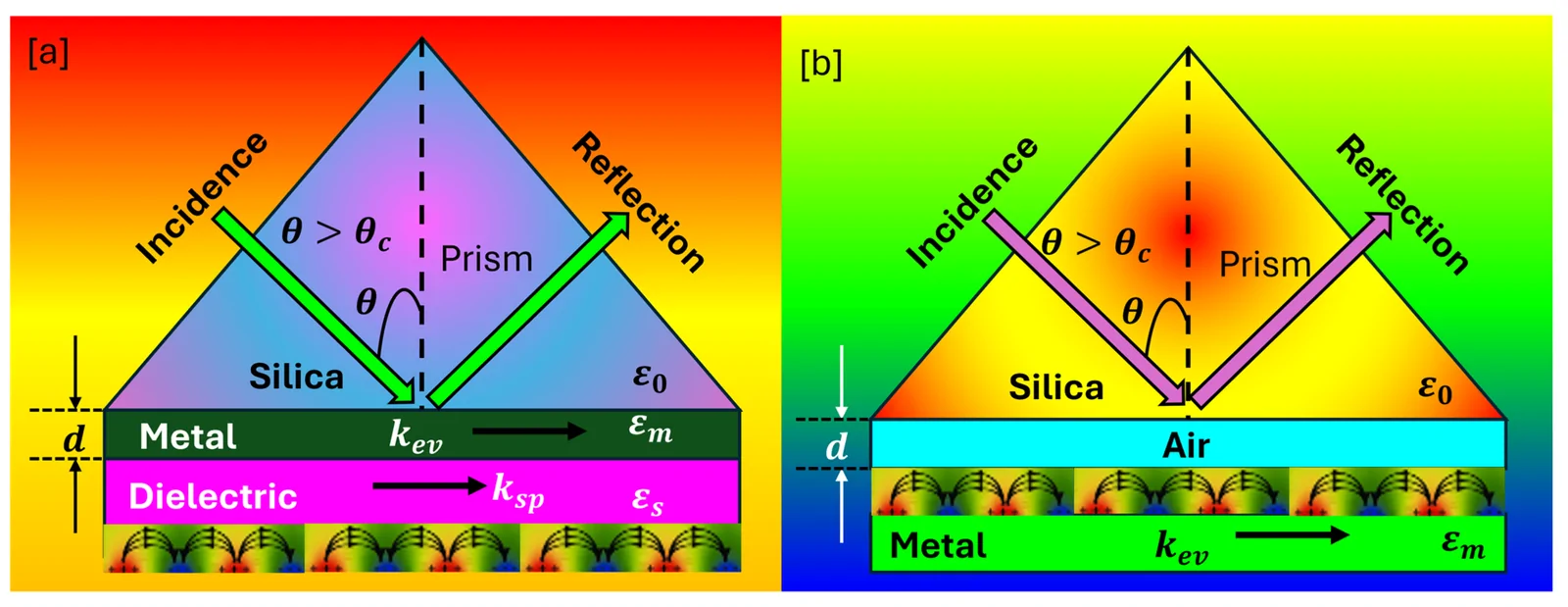
SPR-based configuration. (**a**) Kretschmann configuration; (**b**) Otto configuration.

Photonic crystal fiber (PCF)-based surface plasmon resonance (SPR) biosensors working on the refractive index (RI) variations are extremely sensitive miniature optical devices used to detect variations in analyte by analyzing a red shift and blue shift within the sensor operational wavelength [1]. These sensors are used nowadays in several applications related to biomedical, chemical, biochemical, environmental monitoring, food quality inspection, etc. [2]. These miniature devices are fabricated on the PCF platform due to its unique property to control the light propagation within the fiber body. In addition, PCF has the flexibility to alter geometrical design to achieve highly sensitive parameters. PCF has several advantages like dispersion properties, nonlinearity, controllable birefringence, mode behavior, geometrical design variation, etc. Initially, a Kretschmann configuration was the earliest and most widely used approach for implementing SPR sensors. In this process, a thin metallic layer, typically of gold (Au), or silver (Ag), is directly coated onto the base of a high-RI prism, allowing incident light to interact through the prism and with the metal–dielectric interface [3,4]. At a specific angle of incidence, known as the resonance angle, the momentum of the incident photons matches that of surface plasmons, resulting in the excitation of the collective electron oscillations at the metal surface, resulting in a sharp dip in the reflected light intensity. This dip is extremely sensitive to changes in the RI of the medium, making it useful for sensing applications [5]. However, despite its effectiveness, the Kretschmann setup suffers from several practical limitations. The requirement for a prism-based optical setup makes the system bulky and less portable, while precisely controlling the incident angle and mechanical components such as rotation stages, alignment mounts, etc. [6]. Additionally, careful alignment of the other optical elements, such as the light source and detector, is essential for accurate measurements. These factors increase system complexity, cost, and sensitivity to external disturbances, thereby limiting their suitability for compact, field-deployable, or real-time sensing applications compared to modern fiber-based SPR sensors. Similarly, Au nano cone enables SPR; metal–insulator–metal (MIM) waveguide coupled with Ag cylinder and square cavity can also be used in SPR sensing applications [7,8]. Figure 1a,b represents the schematic of the Kretschmann and Otto configurations, respectively, that were used initially for the SPR-based sensing applications [9].

Fiber-based SPR sensors began to be developed due to advantages like small size, small sample volume, remote sensing ability, etc. The working of the PCF–SPR sensor follows the coupling of surface plasmon polariton (SPP) and leaky core mode at a particular wavelength (λ) known as the resonance wavelength (RW). At this wavelength, the real and imaginary parts of the effective mode index become identical [10]. The RW is different for distinct analytes and varies based on the change in RI of the analyte. Another important aspect of the PCF–SPR sensor is the plasmonic material. There are different types of plasmonic materials used in these sensors to date. These materials can be generally distinguished into two categories: conventional materials and emerging materials. Conventional materials include Ag, titanium dioxide ($TiO_{2}$), copper (Cu), Au, aluminum (Al), graphene, etc. [11,12]. These materials have been used in SPR sensors since the introduction of the SPR theory. Emerging materials include magnesium fluoride ($MgF_{2}$) [13], transparent conducting oxides (TCOs) [14], transition metal dichalcogenides (TMDs) [15], black phosphorus [16], metal carbides [17], nitrides [18], and MXenes [19], etc. Every material possesses some merits as well as shortcomings, like Au is widely recognized as a stable and efficient plasmonic material that assists in the generation of the SPR and is chemically inert too, but at the same time it gets oxidized quickly. Thus, it is suggested by experts to use a dual coat of Au and $TiO_{2}$ for enhanced durability. This combination allows sensors to operate effectively for a longer duration of time.

PCF–SPR sensors can be fabricated using different design techniques, which include internal metal deposition (IMD), external metal deposition (EMD), D- shaped PCF, etc. [20,21]. In addition, several biomedical applications for which PCF–SPR sensors can be used are cancer detection [22], glucose monitoring [23], blood component analysis [24], heavy metal analysis [25], COVID virus identification [26], pregnancy detection [27], etc. Thus, in this investigation, a model of a EMD-based PCF–SPR sensor having a coating of Au and $TiO_{2}$ is presented, which is specifically designed to investigate the RI ranging from 1.33 to 1.37 RIU representing cancerous cellular environments [28,29]. Typically, normal cells exhibit RI values in the range of 1.33–1.34, while cancerous cells demonstrate higher RI approximately 1.35–1.37, and beyond due to increased intracellular protein content, altered morphology, and enhanced biochemical activity. Consequently, monitoring RI variations within this range enables the detection of cancer-associated changes in biological samples. Several other methods like surface-enhanced Raman scattering (SERS) [30], actuation of micro/nanorobots [31], can also play a significant role in cancer detection.

## 2. Geometrical Description of the Sensor Model

A EMD-based two-dimensional (2D) model of the PCF–SPR sensor is presented in this section and the RI range of 1.33–1.37 RIU is investigated. The representative RI values of 1.33, 1.34, 1.35, 1.36, and 1.37 are used to model water, normal urine, breast cancer-related blood plasma, leukemia-related white blood cells (WBCs), and prostate cancer-related to urine extracellular vesicles, respectively [21,22,23]. Furthermore, a step size of 0.01 is used to design the sensor model. The geometry of the PCF–SPR sensor consists of two dimensions of air holes arranged in a manner that a tetra core is generated within the PCF. The dimensions of the air holes are $d_{1}=1.50\;\mu m$ and $d_{2}=1.20\;\mu m$, respectively. The pitch (Λ) is represented by 2.25 μm. Coating of plasmonic materials Au and $TiO_{2}$ are applied to the sensor surface using the chemical vapor deposition (CVD) technique. The thickness of the Au and $TiO_{2}$ layer is optimized through a parametric investigation by varying its thickness as 40 nm, 45 nm, and 50 nm for Au and 80 nm, 85 nm and 90 nm for $TiO_{2}$, respectively. The resulting resonance characteristics, confinement loss (CL), wavelength sensitivity (WS), and amplitude sensitivity (AS) are analyzed for each case, and it was observed that 45 nm Au and 85 nm $TiO_{2}$ layers provided the most favorable sensing performance. Therefore, the optimized thickness of plasmonic material selected in the sensor model for Au and $TiO_{2}$ are 45 nm and 85 nm, respectively. An analyte sensing layer is used to flow various cancerous cells having a thickness of 2.25 μm; finally, a perfectly matched layer (PML) of thickness 2.50 μm is installed over the sensing layer to keep the reflections within the sensor body.

All numerical analyses of the sensor are performed using COMSOL Multiphysics 6.2 based on the finite element method (FEM). A 2D electromagnetic wave frequency domain (ewfd) solver, wave optics module is used to investigate the modal characteristics of the proposed PCF–SPR sensor.

Background material used in the sensor model is fused silica ($SiO_{2}$) those RI is expressed by the “Sellmeier equation” represented by Equation (1) [32].

(1) $$n\left(\lambda \right)=\sqrt{1+\frac{a_{1}\lambda^{2}}{\lambda^{2}-b_{1}}+\frac{a_{2}\lambda^{2}}{\lambda^{2}-b_{2}}+\frac{a_{3}\lambda^{2}}{\lambda^{2}-b_{3}}}$$

where $a_{1}$, $a_{2}$, $a_{3}$, $b_{1}$, $b_{2}$, and $b_{3}$ are known as Sellmeier coefficients, but their values differ for different materials since we are using fused $SiO_{2}$ as the background material, then the expression of the Sellmeier equation is expressed by Equation (2) [33].

(2) $$n_{SiO_{2}}\left(\lambda \right)=\sqrt{1+\frac{0.696166300\lambda^{2}}{\lambda^{2}-4.67914826}+\frac{0.407942600\lambda^{2}}{\lambda^{2}-1.35120631\times 10^{-2}}+\frac{0.897479400\lambda^{2}}{\lambda^{2}-97.9340025}}$$

The RI of Au is expressed by the “Drude Lorentz model,” which is represented by Equation (3) [34,35].

(3) $$\epsilon \left(\omega \right)=\epsilon_{\infty }-\frac{\omega D^{2}}{\omega \left(\omega +j\gamma_{D}\right)}-\frac{∆\varepsilon \Omega_{L}^{2}}{\left(\omega^{2}-\Omega_{L}^{2}\right)+j\Gamma_{L}\omega }$$

where $\epsilon_{\infty }$ is defined as frequency permittivity and is equal to 5.9673; optical angular frequency is expressed by ω, $\omega_{D}$ is the plasma frequency, and the expression $\omega_{D}/2\pi =2113.6$ THz, damping frequency is expressed via $\gamma_{D}$ and expression $\gamma_{D}/2\pi =15.92\;THz$. The term ∆ε is called the weighing factor and is equal to 1.09. The strength of the oscillator is denoted by $\Omega_{D}/2\pi$ and is equal to 650.07 THz, finally the spectral width of the Lorentz oscillator is expressed by $\Gamma_{L}/2\pi =104.86\;THz$ [33,36].

In the proposed sensor, an additional layer of $TiO_{2}$ is used as an adhesive layer to anchor the plasmonic metal onto the fiber surface. $TiO_{2}$ layer enhances the interaction between the fundamental guided mode and the surface plasmon modes. Additionally, the $TiO_{2}$ coating enables the tuning of the SPR sensor’s operational wavelength toward the near-infrared region [37]. In comparison to the visible spectrum, the evanescent field in the near-infrared region exhibits greater penetration depth, thereby significantly improving the sensitivity of the SPR sensor [33,38].

The RI of $TiO_{2}$ is expressed by Equation (4) [38]. Further details about the terminology can be obtained from [38].

(4) $$n_{TiO_{2}}^{2}=5.913+\frac{0.2441}{\lambda^{2}-0.0803}$$

**Figure 2:**
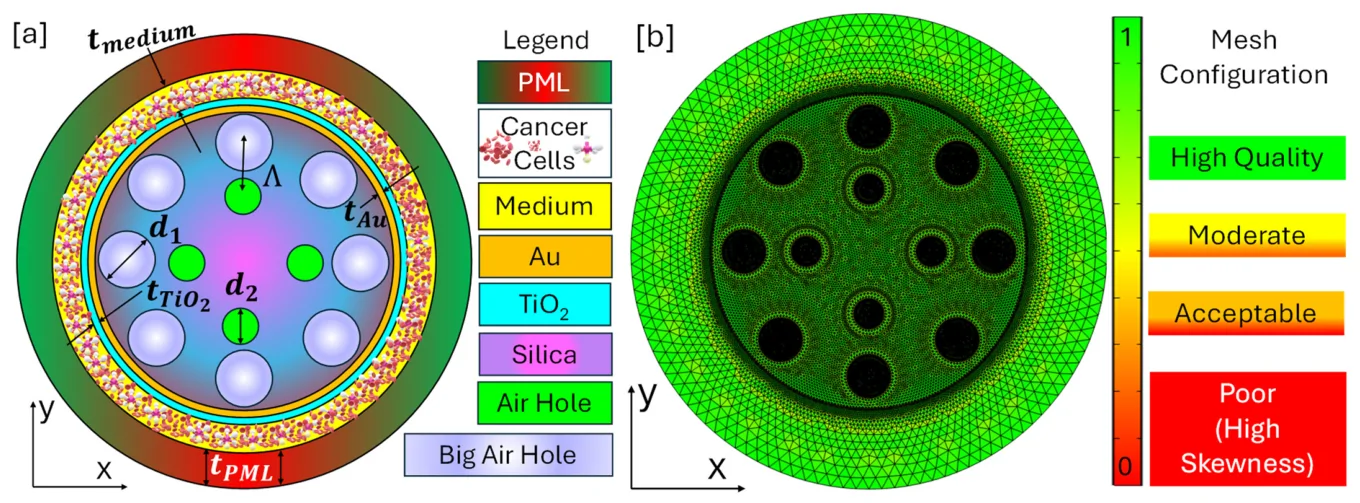
(**a**) Structural layout of the proposed sensor configuration; (**b**) mesh configuration of the proposed sensor showing adaptive triangular elements and high mesh quality.

Figure 2a represents the structural layout of the sensor model with classification of the various domains, air hole geometries, plasmonic material thickness, sensing channel (medium), and PML boundary.

A domain-specific non-uniform free triangular mesh is employed to accurately resolve the optical field distribution in different regions of the sensor model. For the fused silica, a custom mesh is adopted with a maximum element size of 0.1 μm, a maximum growth rate of 1.0, a curvature factor of 0.2, and a narrow region resolution of 1.0. The air-hole regions are discretized using a finer custom mesh with a maximum element size of 0.025 μm, a minimum element size of $3.05\;\times \;10^{-6}\;\mu m$, a maximum growth rate of 1.0, a curvature factor of 0.1, and a narrow region resolution of 0.1. The Au plasmonic layer is meshed using a predefined finer mesh configuration having a maximum element size of 0.564 μm, a minimum element size of 0.00191 μm, a maximum growth rate of 1.25, a curvature factor of 0.25, and a narrow region resolution of 1.0. Similarly, the $TiO_{2}$ adhesion layer and analyte region are discretized using predefined extra-fine mesh settings with a maximum element size of 0.305 μm, minimum element size of 0.00114 μm, maximum growth rate of 1.2, a curvature factor of 0.25, and a narrow region resolution of 1.0. The PML surrounding the sensing region is meshed using a custom mesh with a maximum element size of 0.5 μm, minimum element size of $5.05\;\times \;10^{-5}\;\mu m$, maximum element growth rate of 1.5, curvature factor of 0.4, and narrow region resolution of 5.0. A free triangular mesh is used throughout the computational domain, while a 2.50 μm thick- PML is employed to absorb outgoing electromagnetic waves and eliminate boundary reflections. The adopted mesh strategy ensured accurate evaluation of RW shifts and CL, particularly at the metal–dielectric interface where surface plasmon excitation occurs. Finally, scattering boundary conditions (SBC) are applied at the outer boundary of the PML region. Figure 2b represents the mesh growth rate of the proposed sensor model. A mesh growth rate in the range of 1.3–1.4 is typically employed to ensure a smooth transition between various kinds of mesh elements. This enables the accurate resolution of strong field gradients near the metal–dielectric interface while maintaining computational efficiency.

**Figure 3:**
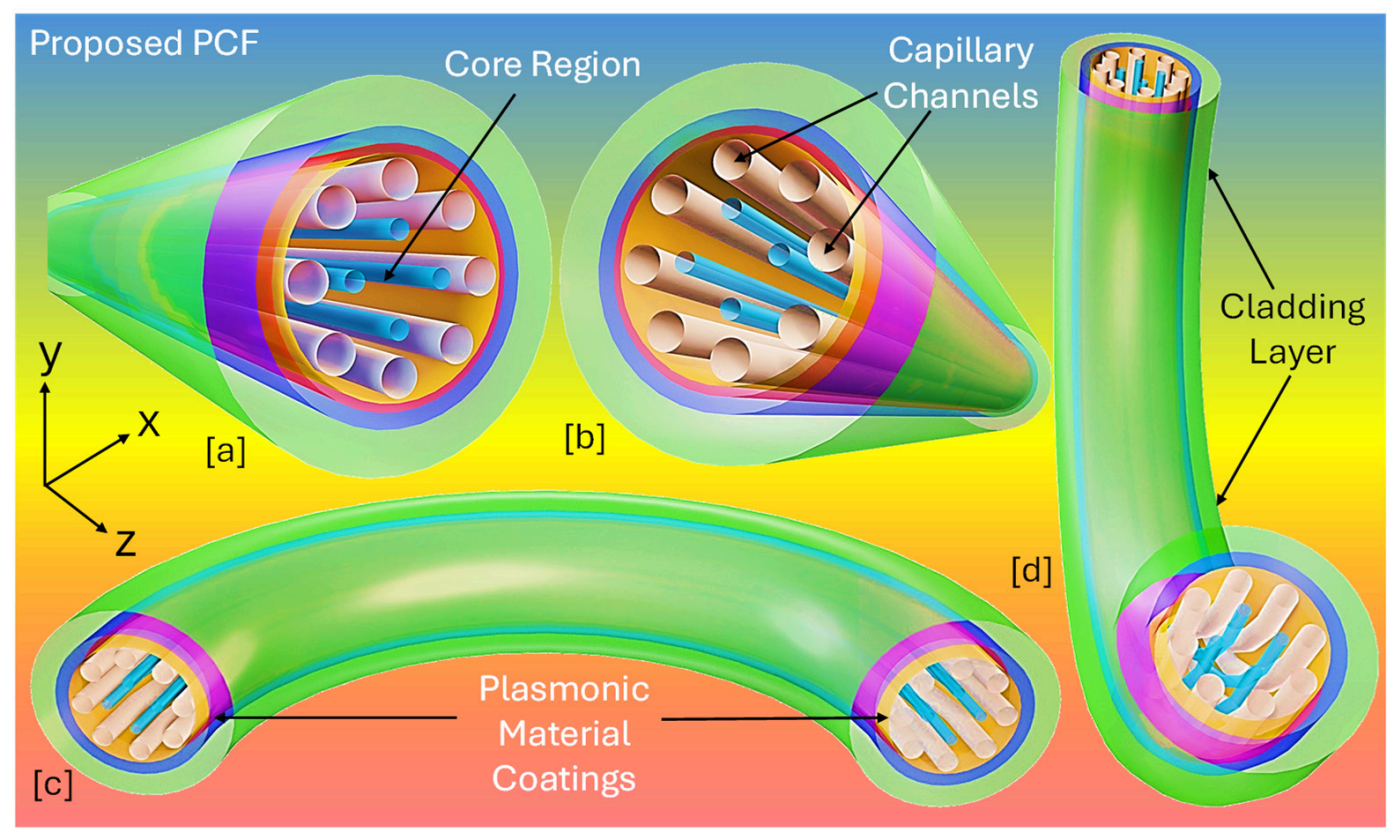
A 3D schematic of the proposed multicore PCF. (**a**) Cross-sectional view showing the arrangement of embedded channels and capillaries; (**b**) perspective view of the internal architecture; (**c**,**d**) 3D views of the proposed PCF from different orientations for enhanced visualization of the plasmonic sensing region and cladding configuration.

The three-dimensional (3D) layout of the proposed sensor features a complex, multi-layered PCF structure specifically engineered for high sensitivity SPR applications as presented in Figure 3. The 3D prototype consists of a specialized internal geometry having a central core surrounded by a symmetrical arrangement of air holes and capillaries. The arrangement of plasmonic material layers within the cladding is responsible for exciting the SPP at the metal–dielectric interface. The structure is created to maintain the functional integrity of the sensor under various physical states, including the following:

- Unbent Configuration: Figure 3a,b show the proposed multicore PCF geometry from cross-sectional and perspective views, highlighting the arrangement of air holes, capillary channels, and plasmonic sensing regions.
- Three-Dimensional Visualization: Figure 3c,d present additional 3D views of the proposed structure from different orientations for improved visualization of the plasmonic coating, cladding layer, and internal architecture.

These versatile features of the PCF suggest it as a robust platform for detecting cancerous fluid, where the interaction between the guided light and the plasmonic material is finely tuned by the fiber’s geometrical parameters and its physical orientation.

Regarding fabrication feasibility, the proposed PCF–SPR sensor can be realized using stack-and-draw or extrusion techniques commonly used for PCF fabrication. The selective deposition of $TiO_{2}$ and Au thin films can be made through methods such as atomic layer deposition (ALD) [39], CVD [40], sputtering [41], or electron-beam evaporation [42]. The realization of the proposed sensor may present several practical challenges, such as maintaining the dimensional accuracy of the proposed tetra-core geometry during the fiber drawing process, achieving uniform and controllable $TiO_{2}/Au$ bilayer thickness, ensuring strong adhesion between adjacent material layers, and selectively coating only the designated sensing region while avoiding undesired material deposition on neighboring surfaces. In addition, slight deviations in the structural parameters introduced during fabrication can influence the phase-matching condition and resonance characteristics. Recent developments in microstructured fiber fabrication, precision-controlled thin-film deposition, and post-processing techniques have significantly improved the manufacturability of several complex PCF-based plasmonic sensor structures. Therefore, the practical realization and experimental development of the proposed biosensor is considered feasible and constitutes an important part of future work.

## 3. Biological Relevance of Selected Refractive Index Values for Cancer Biosensing

The RI of a biological sample is closely associated with its biochemical composition, cellular concentration, and molecular content. Variations in RI can arise due to changes in proteins, cells, extracellular vesicles, metabolites, and other biomolecular constituents present in the sample. Therefore, RI is considered a useful biophysical parameter for the optical characterization of biological fluids and disease-related biomarkers. In this study, the selected RI values ranging from 1.33 to 1.37 are chosen based on the literature-reported data and their biological relevance to represent physiological and pathological conditions. In particular, the RI values of 1.35, 1.36, and 1.37 are associated with blood plasma, WBC-rich samples, and extracellular-vesicle-rich urine samples, respectively, which have been extensively investigated in cancer biomarker studies related to breast, leukemia, and prostate cancers [43,44]. Table 1 summarizes the literature-reported RI values, representative biological samples, and their diagnostic significance in cancer biosensing applications.

The RI value of 1.35 is biologically relevant for breast cancer as it falls within the typical RI range reported for blood plasma. The optical properties of blood plasma are largely governed by the concentration of proteins, lipids, metabolites, and other dissolved biomolecules. In cancer patients, alterations in plasma composition can be caused by circulating tumor-derived molecules, proteins, and extracellular vesicles that may lead to measurable changes in the RI. Consequently, plasma RI has been investigated as a potential biophysical indicator in breast, lung, colorectal, and other cancer-related biomarker studies [43,44].

A RI of approximately 1.36 is commonly associated with samples having a relatively high cellular content, particularly for WBC-rich suspensions. The increase in cellular concentration elevates the overall optical density of the sample, resulting in a higher RI compared with normal plasma. Such RI values are frequently used in hematological investigations where the concentration, morphology, and physiological state of immune cells significantly influence the optical properties of the sample. In clinical settings, elevated cellular content and corresponding RI changes have been linked to leukemia-related abnormalities and immune-cell-associated disorders. Therefore, RI values around 1.36 are particularly relevant for leukemia diagnostics and studies involving abnormal proliferation or activation of WBC [43,44].

A RI value of approximately 1.37 is commonly associated with EV- or exosome-rich biological samples, including certain urinary specimens. Extracellular vesicles are nanoscale membrane-bound particles released by cells and contain proteins, lipids, nucleic acids, and other biomolecular cargos that reflect the physiological state of their parent cells. An increased concentration of EVs and exosomes raises the optical density of the biological fluid, resulting in a measurable increase in RI. In recent years, urinary EVs and exosomes have gained considerable attention as promising non-invasive biomarkers for cancer diagnosis and monitoring, particularly in prostate, bladder, and kidney cancers. Therefore, the RI range around 1.37 is of significant clinical interest for EV-based liquid biopsy applications [43,44].

However, since RI variations are not uniquely disease-specific, practical clinical applications may benefit from combining RI measurements with complementary molecular biomarkers and advanced data-analysis approaches to improve diagnostic specificity and reliability. Table 2 presents a detailed overview of the various cancer types, associated body fluid samples, and relevant biomarkers causing them.

## 4. Optical Mode Profiles of the Proposed Biosensor

The RI range of 1.33–1.37 RIU is investigated in this study for analyzing cancer cells, for potential biomedical sensing applications. An RI value of 1.33 represents water, which serves as a baseline reference [57]. The RI value of 1.34 represents normal human urine. However, any deviations from this level may indicate pathological conditions such as chronic kidney disease or urinary tract infection [58,59]. An RI of approximately 1.35 is associated with blood plasma, where variations may arise due to metabolic and physiological changes, including diabetes mellitus, increased protein concentration, or dehydration [60]. The RI value of 1.36 correspond to WBC, and any changes in this range can be linked to immune-related conditions such as leukemia, systemic infections like Sepsis, or inflammatory responses [61]. A higher RI value of 1.37, particularly in urine containing extracellular vesicles, reflects elevated biomolecular content and may be associated with serious conditions including prostate cancer, renal disorders, and certain neurodegenerative diseases [62].

**Figure 4:**
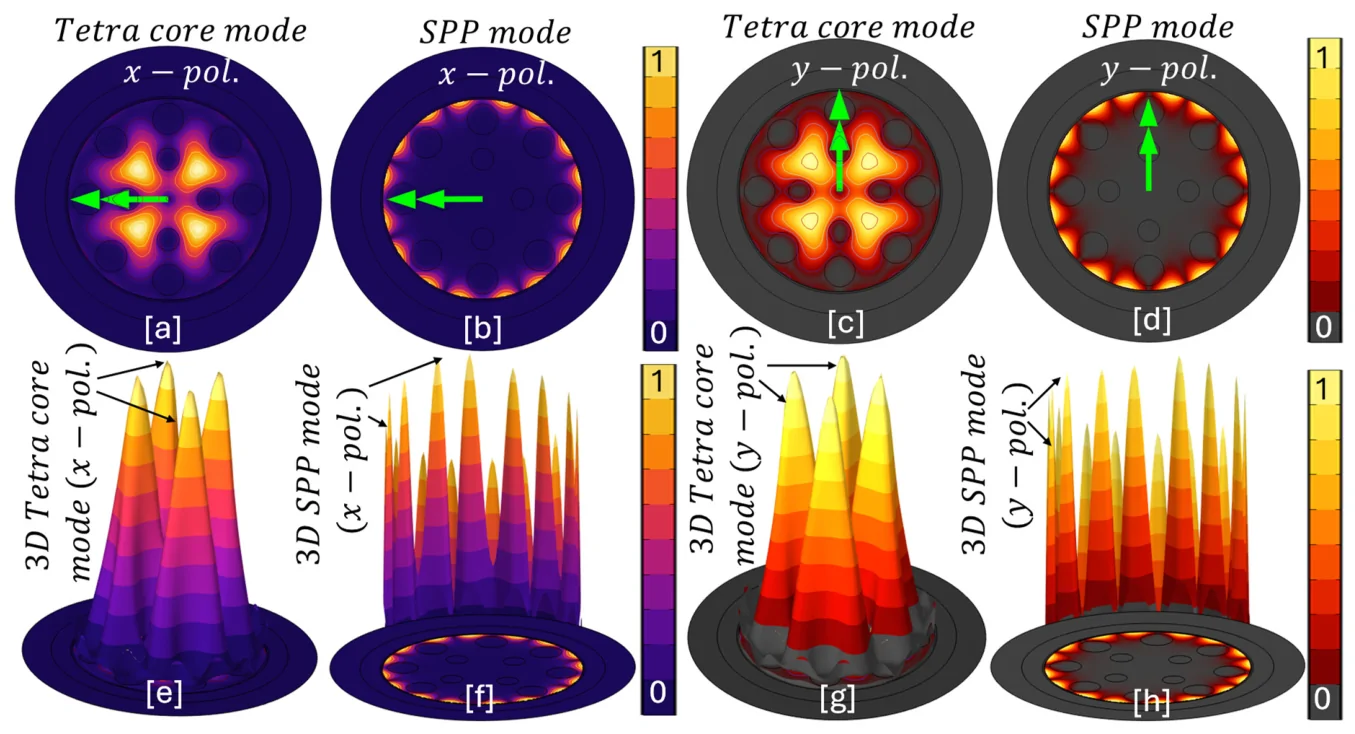
Two-dimensional and 3D mode profiles for the proposed sensor for RI 1.33; 2D mode profiles for x-pol: (**a**) Tetra-core mode; (**b**) SPP mode; y-pol. mode profile; (**c**) tetra-core mode; (**d**) SPP mode. Three-dimensional mode profiles for x-pol: (**e**) tetra-core model; (**f**) SPP mode, y-pol. mode profile; (**g**) tetra-core mode; (**h**) SPP mode.

In this investigation, we have performed dual-mode analysis of the sensor model, which includes both x-pol. (x-polarization) and y-pol. (y-polarization) with the objective of obtaining maximum information from the sensor. The 2D and 3D core mode and SPP mode profiles of the sensor concerning x-pol. and y-pol. are presented in Figure 4.

Figure 4a,b represent the 2D x-pol. Tetra-core mode profile and x-pol. SPP mode profile, Figure 4c,d represent the 2D y-pol. Tetra-core mode profile and y-pol. SPP mode profile, Figure 4e,f represents the 3D tetra-core mode profile and 3D SPP mode profile for x-pol. Finally, Figure 4g,h represent the 3D tetra-core mode profile and 3D SPP mode profile for y-pol. These mode profiles are extracted from the proposed sensor corresponding to RI 1.33. Similarly, mode profiles for other RI values can also be extracted.

When we compare the tetra-core configuration with the single-core [63] and dual-core [64] configuration, the tetra-core fiber [65] offers significantly enhanced sensing performance; this is due to the increased modal interactions and multiple coupling pathways. The presence of four closely spaced cores enables strong supermode formation and efficient coupling between guided modes and the plasmonic interface, which results in higher sensitivity and multiple resonance peaks. Additionally, the tetra-core designs provide greater flexibility in tuning the structural parameters, which allows optimization of birefringence and sensing characteristics. But at the same time, besides these advantages, there are also some challenges, like increased structural complexity, complex fabrication, and regular monitoring.

## 5. Experimental Characterization of Breast Cancer (T-47D), Leukemia (MM6), and Prostate Cancer (DU145) Cell Lines for RI-Based Biosensor

**Figure 5:**
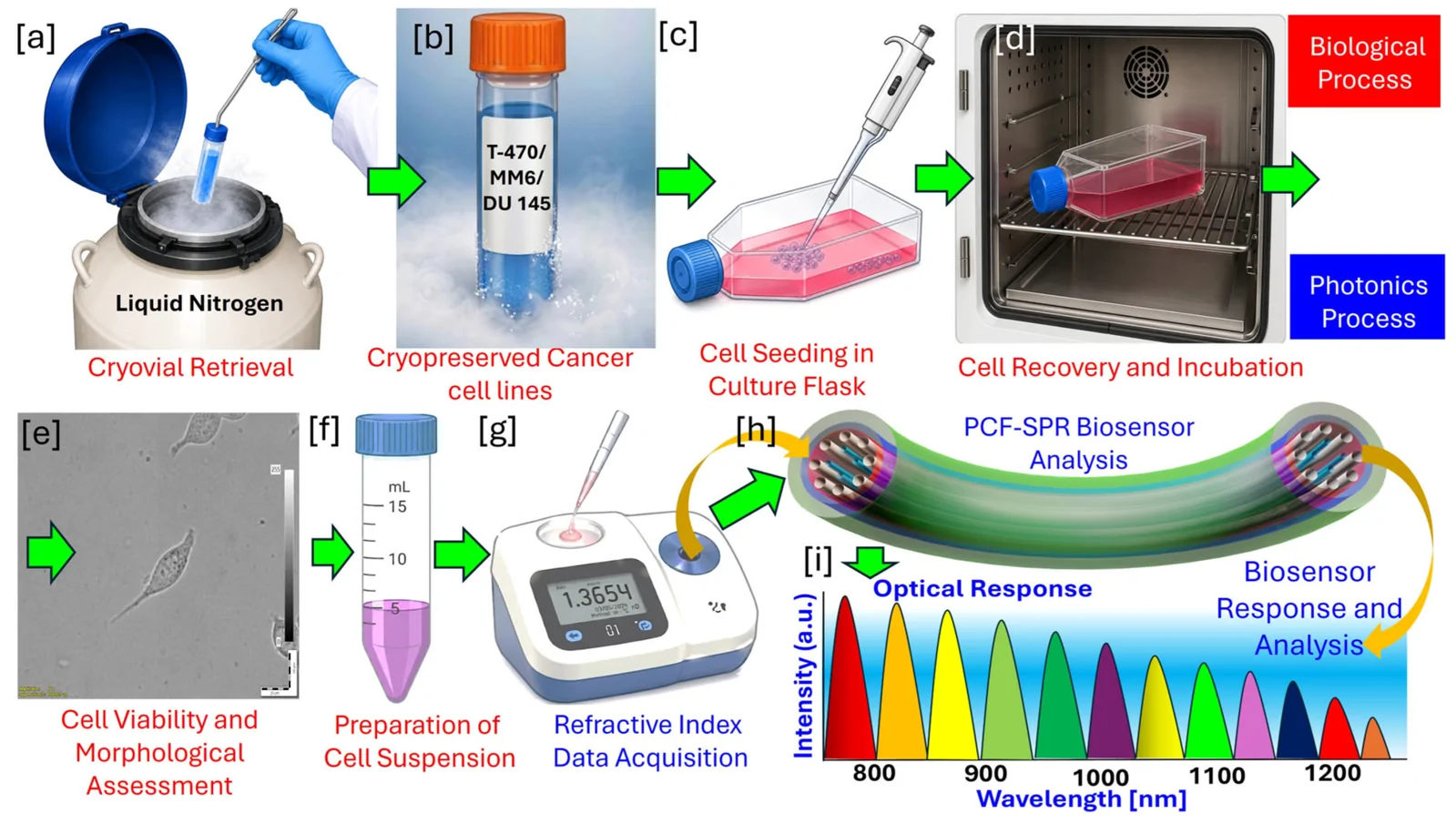
Experimental workflow demonstration using T-47D, MM6, and DU145 cancer cell lines for the proposed PCF–SPR biosensor. (**a**) Retrieval of cryopreserved cell vials from liquid nitrogen storage; (**b**) cryopreserved cancer cell lines used in this study; (**c**) seeding of cells into culture flasks containing appropriate culture medium; (**d**) cell recovery and incubation; (**e**) cell viability and morphological assessment; (**f**) preparation of cell suspension; (**g**) schematic representation of RI determination for cancer cell samples based on literature-reported values; (**h**) PCF–SPR biosensor analysis; (**i**) biosensor response and analysis based on resonance wavelength shifts.

To demonstrate the biological relevance of the presented PCF–SPR biosensor for cancer detection. Distinct cancer cells are cultured under standard cell culture conditions, specifically in a cell-culture flask containing a nutrient-rich growth medium. The cultured cells are analyzed using an optical microscope connected to a computer for microscopic image acquisition and visualization [66]. The microscope is focused on different regions of the culture surface, and representative microscopic images are obtained. The acquired images showed active proliferation of T-47D for breast cancer [67], monomac 6 (MM6) for leukemia [68] and DU145 for prostate cancer cells [69] within the culture medium. The experimental demonstration workflow of the proposed cancer biomarkers is illustrated in Figure 5.

Initially, cryopreserved T-47D, MM6, and DU145 cell lines are retrieved from liquid nitrogen as presented in Figure 5a,b. Then, the cells are transferred into the appropriate pre-warmed supplemented culture medium for cell seeding, as presented in Figure 5c. The cells are then incubated under standard cell culture conditions to facilitate cellular recovery and proliferation, as presented in Figure 5d. Following the incubation, cell viability and morphology are evaluated using microscopic examination to ensure the suitability of the recovered cells for further analysis, as represented in Figure 5e. Following cell viability and morphological assessment, the samples were prepared for RI data acquisition, as illustrated in Figure 5f. Although the RI of cancer cell suspensions can be measured using a digital refractometer, the RI values used in this study are adopted from reported literature data and illustrated in Figure 5g [55,57]. The obtained cell line is then subsequently employed as input parameters for the proposed biosensor as presented in Figure 5h. Finally, the biosensor response for different cancer cell lines is analyzed through RW shifts in the optical transmission spectra as presented in Figure 5i. This will enable the differentiation of breast, leukemia, and prostate cancer cell samples based on their distinct RI characteristics.

**Figure 6:**
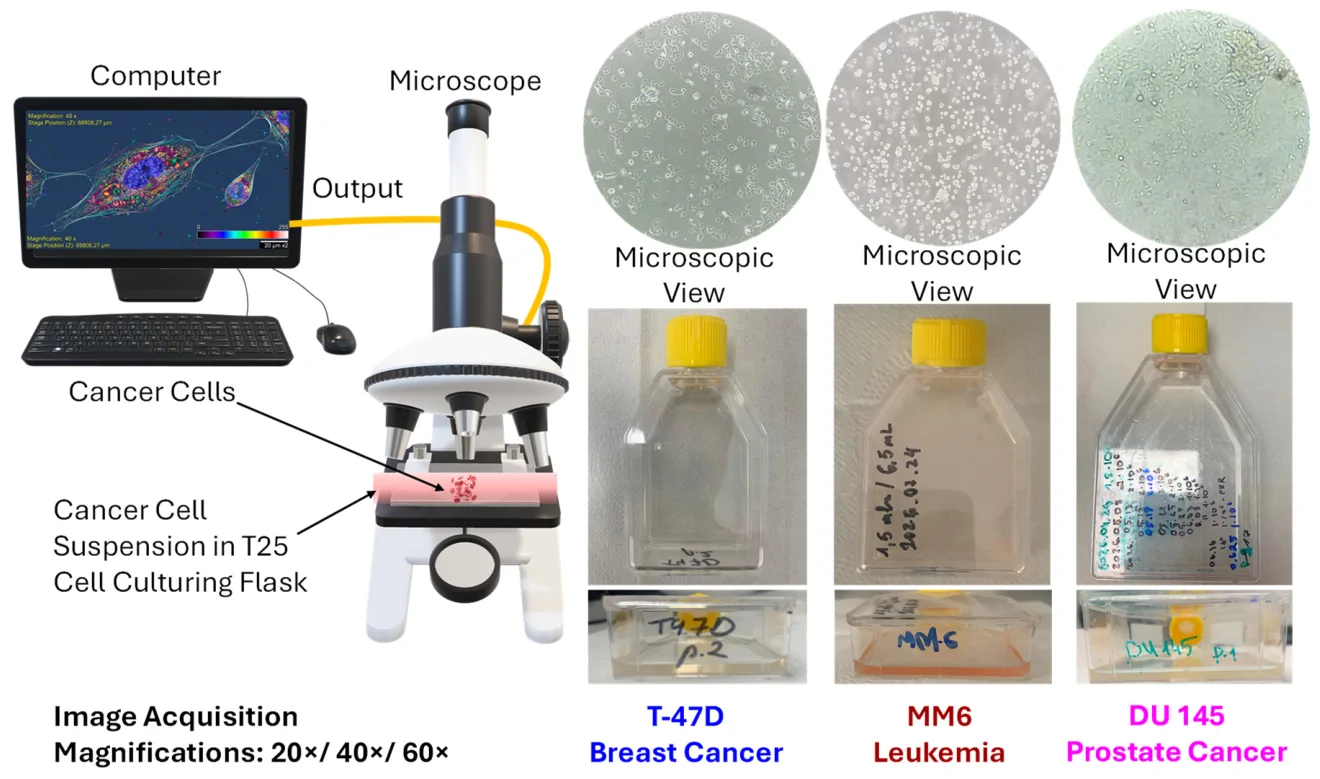
Experimental workflow for cancer cell observation and characterization. T-47D breast cancer, MM6 leukemia, and DU145 prostate cancer cells are cultured and imaged using a computer-assisted optical microscopy system at different magnifications for morphological assessment. Representative microscopic images and culture samples are shown together with the corresponding literature-reported RI values used as input parameters for the proposed biosensor.

Figure 6 illustrates the experimental workflow used for cancer cell observation and characterization. Cancer cells suspended in culture medium are introduced into an optical fluidic chamber mounted on a microscope stage. The microscopic images are captured and transferred to a computer for visualization and morphological assessment of the cells. Three representative cancer cell lines, T-47D, MM6 and DU145, are analyzed. Corresponding microscopic views of the cultured cells are shown along with their RI values of 1.35, 1.36, and 1.37, respectively [70].

T-47D is a widely utilized human breast cancer cell line derived from an invasive ductal carcinoma and is characterized by the expression of estrogen and progesterone receptors, making it a representative model of the luminal-type hormone responsible for breast cancer. These cell lines are extensively employed in studies investigating breast cancer biology, hormone signaling pathways, anticancer drug screening, endocrine resistance, and therapeutic response mechanisms. Morphologically, T-47D cells exhibit an epithelial-like phenotype, typically appearing as rounded to polygonal adherent cells that grow in compact clusters due to pronounced cell–cell adhesion.

**Figure 7:**
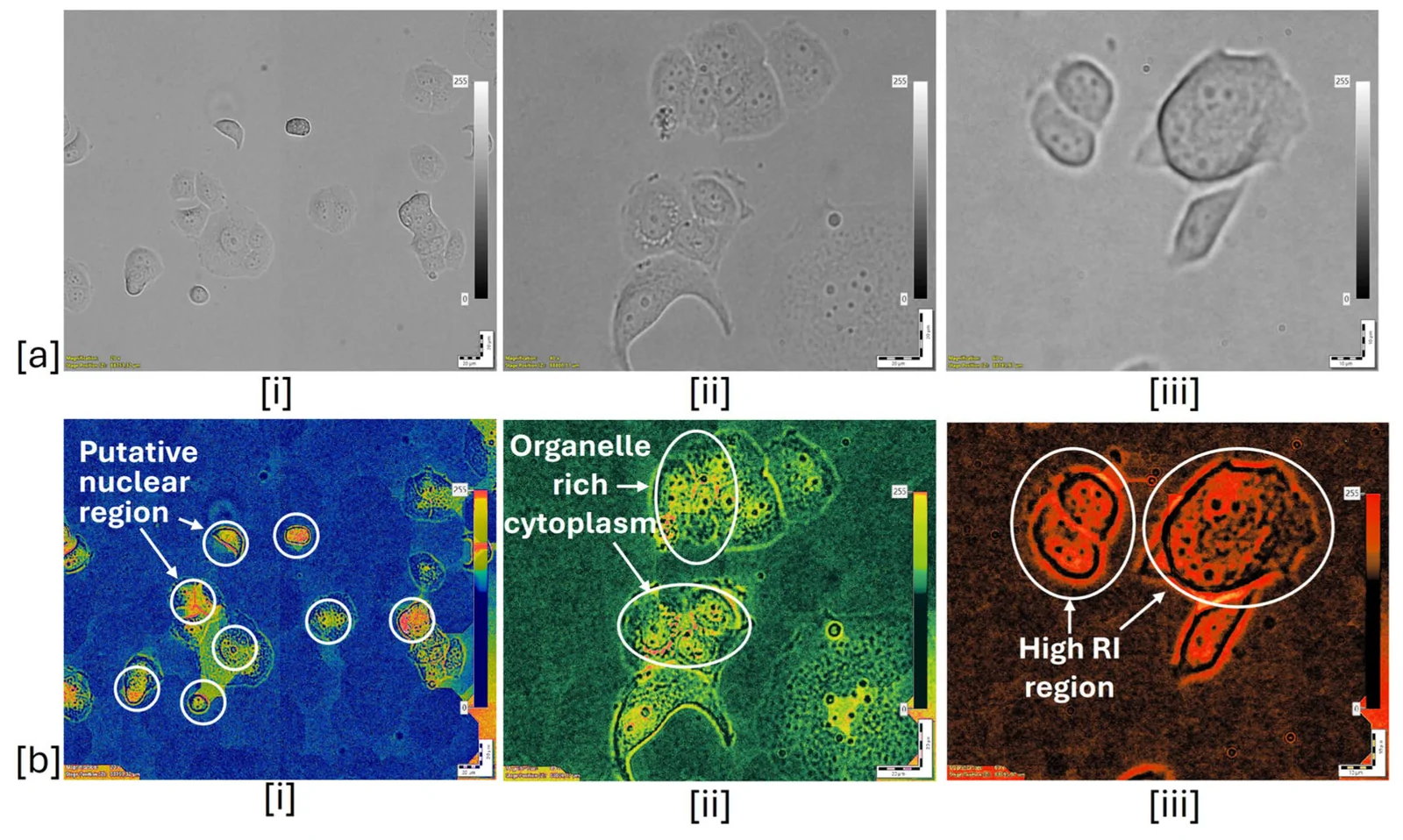
T-47D breast cancer cell (RI = 1.35). (**a**) Grayscale microscopy images (**i**) 20×, (**ii**) 40×, and (**iii**) 60×. (**b**) MATLAB-based enhanced color visualizations captured at magnifications of (**i**) 20× (putative nuclear region), (**ii**) 40× (organelle-rich cytoplasm), and (**iii**) 60× (high-RI region).

The grayscale microscopic images presented in Figure 7a(i–iii) represent the characteristic morphology of T-47D cells at different magnifications (20×, 40×, and 60×). These images reveal the overall cellular distribution, colony organization, and individual cell morphology, while higher magnification images represent improved visualization of cell boundaries, cellular contours, and intracellular texture. The observed clustering behavior and cohesive growth pattern are consistent with the epithelial origin of the T-47D cell line. MATLAB R2022b-based color enhancement is performed through intensity-based contrast enhancement and pseudocolor mapping to improve the visual discrimination of the cellular structures, as represented by Figure 7b(i–iii). The enhanced visualizations facilitate clear identification of the cell boundaries, morphological heterogeneity, and the spatial distribution without affecting the underlying biological features. This enhancement is particularly useful for qualitative assessment, image segmentation, and feature extraction, as it highlights subtle variations in pixel intensity caused by cellular morphology and structural organization. Overall, these images present the typical epithelial morphology, clustered growth behavior, and high RI region of 1.35 that is associated with T-47D breast cancer cells [70]. The RI value of 1.35, reported in the literature for breast cancer, is used as an input parameter in the proposed PCF–SPR biosensor.

**Figure 8:**
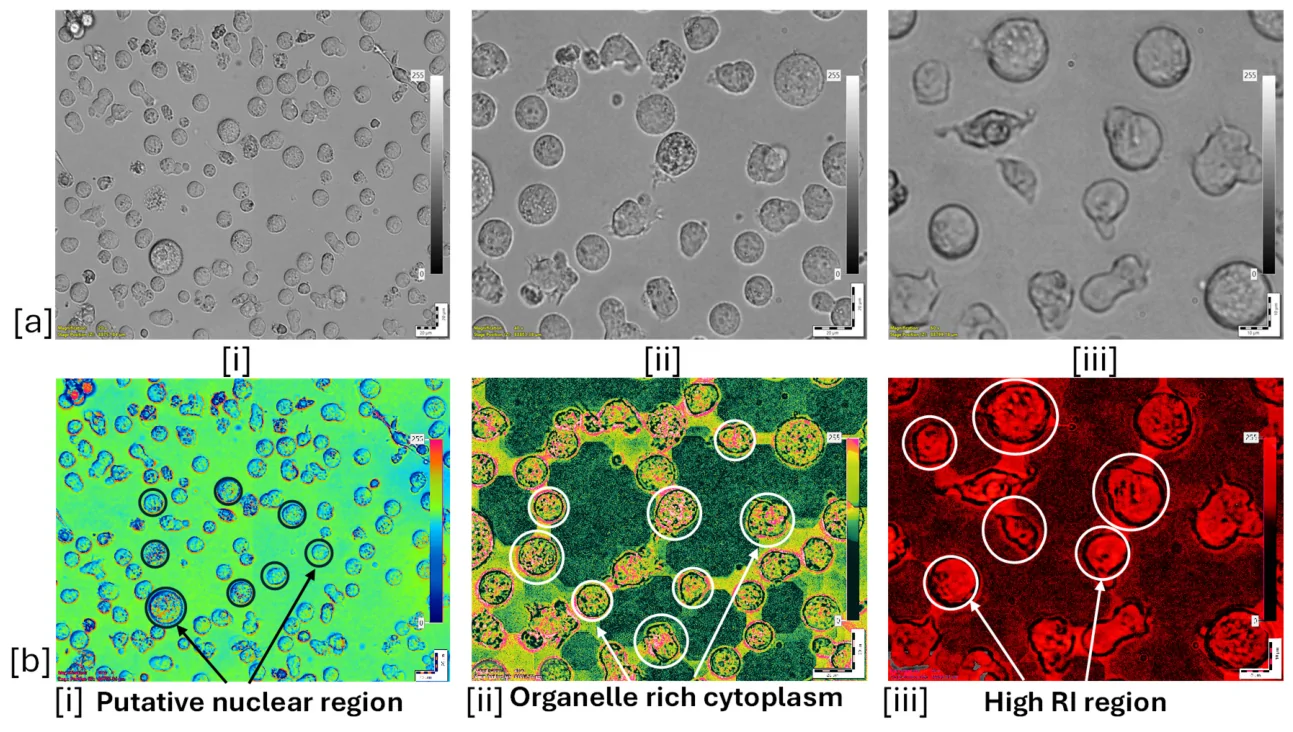
MM6 leukemia cells (RI = 1.36). (**a**) Grayscale microscopic images acquired at (**i**) 20×, (**ii**) 40×, and (**iii**) 60×. (**b**) Corresponding pseudocolor-enhanced representations highlighting the intracellular RI heterogeneity, including (**i**) putative nuclear regions, (**ii**) organelle-rich cytoplasmic compartments, and (**iii**) high-RI regions. The images reveal the characteristic spherical morphology of MM6 suspension cells and provide qualitative support for RI-based cancer cell characterization.

MM6 leukemia cells are used to continuously proliferate in suspension culture and are characterized by their predominantly spherical morphology and relatively uniform cellular distribution pattern. The grayscale microscopic images of the MM6 leukemia cell line are acquired at 20×, 40×, and 60× magnifications and are presented in Figure 8a(i–iii) in original form. Figure 8b(i–iii) presents MATLAB-based pseudocolor enhancement for improved visualization. At lower magnification (20×), the overall population density and distribution of the suspension cells can be readily observed, whereas at the higher magnifications of 40× and 60×, enhanced visualization of individual cellular morphology, cell boundaries, and intracellular features is expressed. The images reveal characteristic round leukemia cells with noticeable intracellular heterogeneity and variable optical contrast. The pseudocolor-enhanced images highlight the putative nuclear regions, organelle-rich cytoplasmic compartments, and localized high-RI domains that indicate the spatial variations in intracellular biomolecular concentration and mass density. The observed morphology of the images confirms successful cell recovery, viability, and maintenance under the standard culture conditions. Following morphological characterization, the MM6 leukemia cells are selected for biosensor demonstration studies. The RI value of 1.36, reported in the literature for blood cancer associated with leukemia, is subsequently used as an input parameter in the proposed PCF–SPR biosensor model [70].

**Figure 9:**
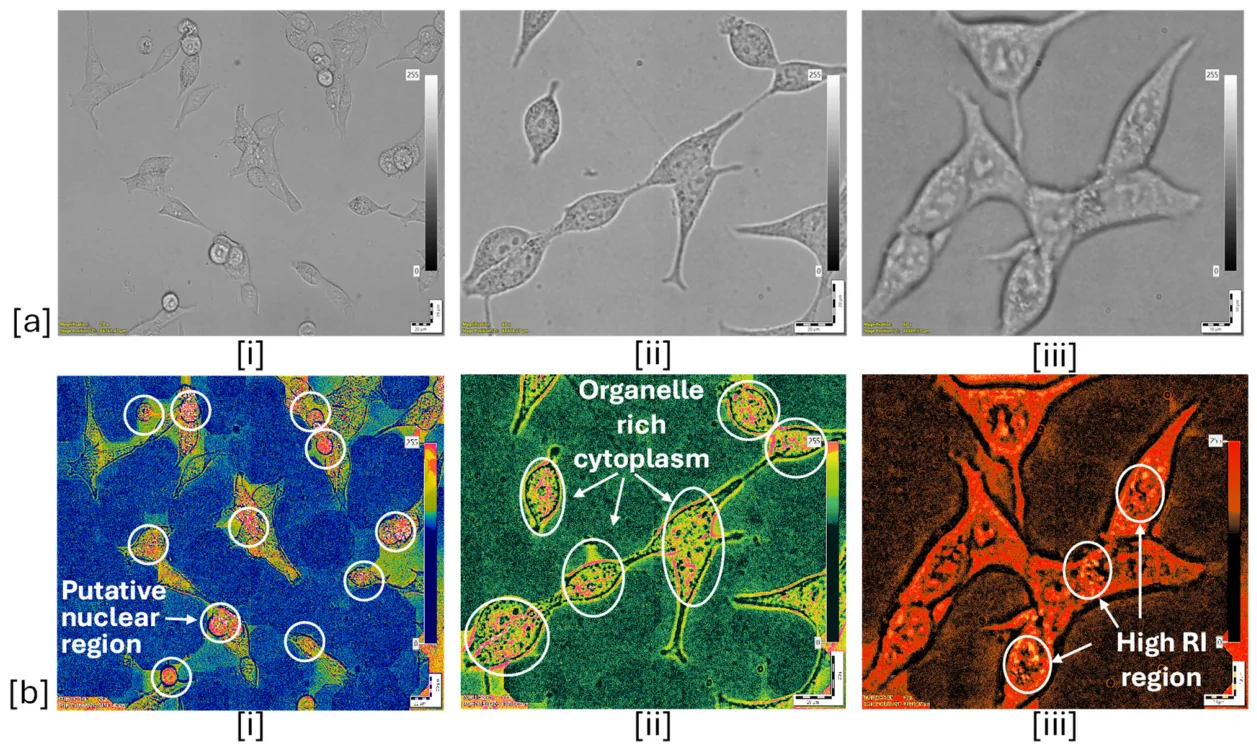
DU-145 prostate cancer cell (RI 1.37). (**a**) Grayscale microscopic images acquired at magnifications of (**i**) 20×, (**ii**) 40×, and (**iii**) 60×, illustrating the morphology and distribution of DU-145 cells. (**b**) Corresponding pseudocolor-enhanced representations of (**i**) 20×, (**ii**) 40×, and (**iii**) 60× images are generated to improve visualization of cellular morphology, boundaries, and intracellular features. The images reveal the characteristic adherent morphology of DU-145 cells and provide qualitative morphological support for the RI-based cancer sensing approach.

Prostate cancer cells continuously release extracellular vesicles (EVs), exosomes, proteins, nucleic acids, and metabolic products into the surrounding medium. These secreted biomolecules can alter the effective RI of the biological environment of the cell. The grayscale microscopic images of the DU145 prostate cancer cell line are obtained at 20×, 40×, and 60× magnifications and are presented in Figure 9a(i–iii). Figure 9b(i–iii) represents the pseudocolor-based images for better visualization. These cells exhibit a characteristic adherent morphology with distinct cellular boundaries and elongated spindle-like structures. At lower magnification (20×), the overall cellular distribution and population density of the cells can be observed, while higher magnifications of 40× and 60× provide enhanced visualization of the individual cell morphology, cellular contours, and intracellular features. The observed morphological characteristics confirm the successful cell recovery, viability, and growth under the standard culture conditions. Following morphological assessment DU145 prostate cancer cells are selected for biosensor demonstration. The RI of 1.37, reported in the literature for prostate cancer, is used as an input parameter in the proposed PCF–SPR biosensor [70].

## 6. Performance Analysis of the Proposed Sensor at the Optimum Plasmonic Thickness

The sensing ability of a PCF–SPR sensor for distinguishing various analytes, chemicals, biochemicals, body fluid samples, and cancer cells is determined using several performance parameters. One among the key parameters is CL, which is generally expressed in dB/cm. CL represents the amount of optical power that is lost from the guided mode due to coupling with surface plasmons at the metal–dielectric interface. A higher CL at the resonance condition indicates stronger interaction between the evanescent field and the investigated analyte, which generally improves sensing performance. It is expressed by Equation (5) [71].

(5) $$CL\;(\alpha )=8.686\times ko\times imag\left(neff\right)\times 10^{4}\;[dB/cm]\;$$

where ko is the wave number and is equivalent to 2π/λ, $imag\left(neff\right)$ is the imaginary part of the effective RI.

Another important parameter is WS, which is measured in nm/RIU, this parameter defines how much the RW shifts in response to a unit change in RI of the surrounding medium. A larger wavelength shift for a small RI variation indicates higher sensitivity of the sensor. It is evaluated using a wavelength interrogation technique and is expressed by Equation (6) [72].

(6) WS=∂λpeak ∆λna[nm/RI]

where $\partial \lambda_{peak}$ represents the change in the RW of the two consecutive analytes and $∆\lambda_{na}$ represents the change RI values of two successive analytes.

AS of the sensor model describes the variation in the transmitted or loss spectrum intensity with respect to changes in RI at a fixed wavelength, and it is useful for intensity-based sensing approaches. It is expressed by Equation (7) [73].

(7) AS=−(1 αλ, na)×(∂α(λ, na) ∂na) [RIU−1]

where $\alpha \left(\lambda ,\;na\right)$ represents the CL of the fundamental mode and ∂α(λ, na) represents the change developed in the CL of two successive analytes.

Sensor resolution (SR) is measured in RIU and represents the smallest detectable change in RI that the sensor can measure. A lower value of SR indicates a more precise and efficient sensing system. It can be expressed by Equation (8) [74].

(8) SR=∂na×(∂λmin/∂λpeak)[RIU]

where ∂λ min is the minimum spectral resolution, and usually its value is considered as 0.1 nm. The minimum detectable shift of 0.1 nm is assumed according to the spectral resolution of most of the commercially available optical spectrum analyzer (OSA). ∂λpeak represents the change in the RW of two consecutive analytes and ∂na represents the change in the RI of the analytes.

Later, the relationship between RI and RW is established through a calibration or fitting curve; the resulting calibration equation can then be used to estimate unknown RI values from measured RW shifts.

Figure of merit (FOM) is an important performance parameter that calculates overall sensing capability by considering both WS and resonance line width. It is expressed by Equation (9) [75].

(9) $$FOM=WS/FWHM[{RIU}^{-1}]$$

where WS represents the wavelength sensitivity (nm/RIU) and FWHM denotes the full width at half maximum (nm) of the resonance loss spectrum.

Together, these parameters collectively determine the overall performance and reliability of a PCF–SPR sensor for practical sensing applications.

### Sensor Simulation Results at the Optimum Thickness of Plasmonic Material

**Figure 10:**
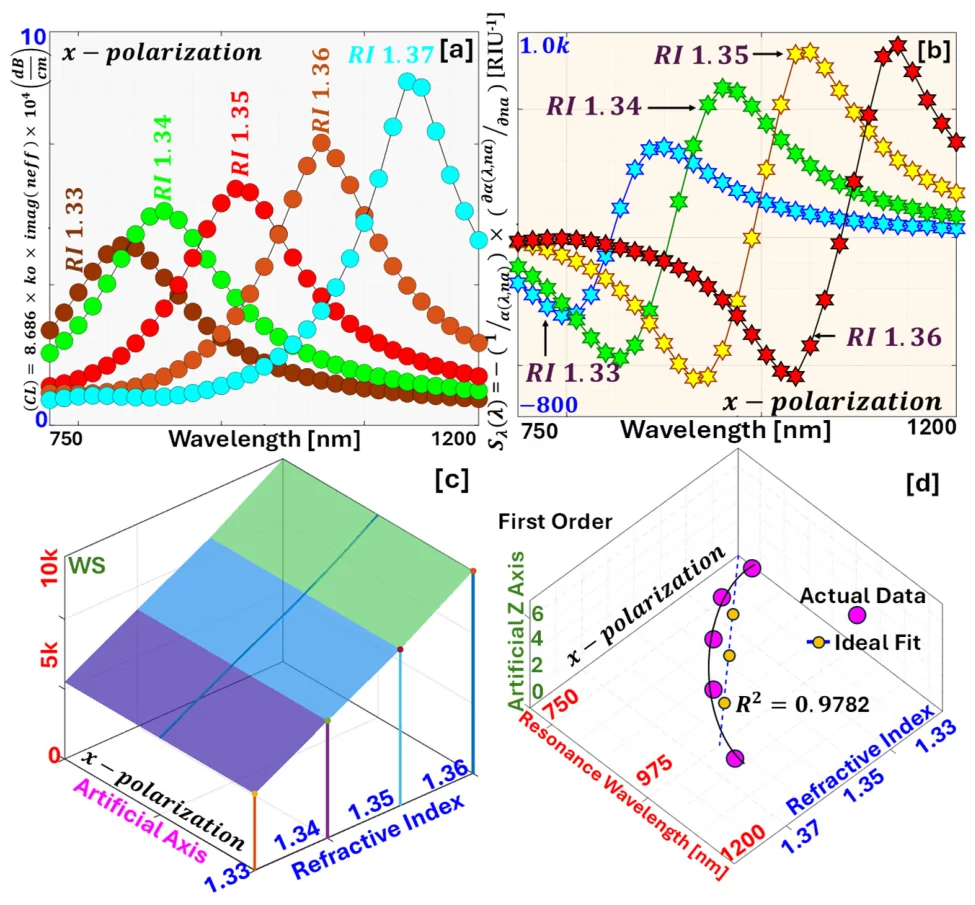
Sensing behavior of the sensor model for x-pol. (**a**) CL; (**b**) AS; (**c**) WS; (**d**) relationship between RW and RI.

In this section, we have investigated the sensor sensing behavior at the optimum thickness of plasmonic materials, i.e., 45 nm Au  and 85 nm $TiO_{2}$ for both x-pol. and y-pol., respectively. The numerical investigations are carried out in the wavelength range of 750–1200 nm with a wavelength increment of 15 nm. The simulation results corresponding to x-pol. of the sensor model are presented in Figure 10.

Figure 10a represent CL of 4.1053, 4.5303, 5.4944, 7.1458 and 9.2657 dB/cm for RI 1.33 (water), 1.34 (urine sample-normal), 1.35 (T-47D breast cancer), 1.36 (MM6-leukemia) and 1.37 (DU145 prostate cancer) respectively corresponding to x-pol. at RW of 834.06, 870.09, 950.48, 1035.15, and 1132.84 nm respectively. Figure 10b represents AS of 303.834, 471.384, 550.377 and $623.182\;{RIU}^{-1}$ for water, urine sample, T-47D breast cancer and MM6 leukemia, respectively, corresponding to x-pol. The WS of 3603, 8039, 8467 and 9769 nm/RIU is obtained for water, urine sample, T-47 D breast cancer, and MM6 leukemia, respectively, corresponding to x-pol. as represented in Figure 10c. Figure 10d represents the first-order linear relationship between the RW and RI. The linear relationship is expressed by Equation (10).

(10) $$f\left(x\right)=p_{1}\left(x\right)+p_{2}$$

where $p_{1}=7626,$ and $p_{2}=-9331$. The goodness of fit is expressed by parameters like sum of squared error (SSE) = 1295, root mean square error (RMSE) = 20.78, adjusted $R^{2}=0.971,$ and the coefficient of determination $R^{2}=0.9782,$ representing good fitting between the sensor parameters corresponding to x-pol. The minimum detectable shift of 0.1 nm is assumed according to the spectral resolution of most of the commercially available OSA while calculating SR for the proposed sensor. The SR of $2.7755\times 10^{-5}$, $1.12439\times 10^{-5}$, $1.1811\times 10^{-5}$, and $1.0236\times 10^{-5}$ RIU is obtained for water, urine sample, T-47D breast cancer and MM6 leukemia, respectively, for x-pol. Finally, the FWHM of 166.04, 187.62, 183.79, and 162.34 nm is obtained, resulting in FOM of 21.69, 42.84, 46.06, and $60.17\;{RIU}^{-1}$ for water, urine sample, T-47D breast cancer, and MM6 leukemia, respectively, corresponding to x-pol.

Table 3 summarizes the calculated sensing parameters of the proposed biosensor for x-pol. conditions.

**Figure 11:**
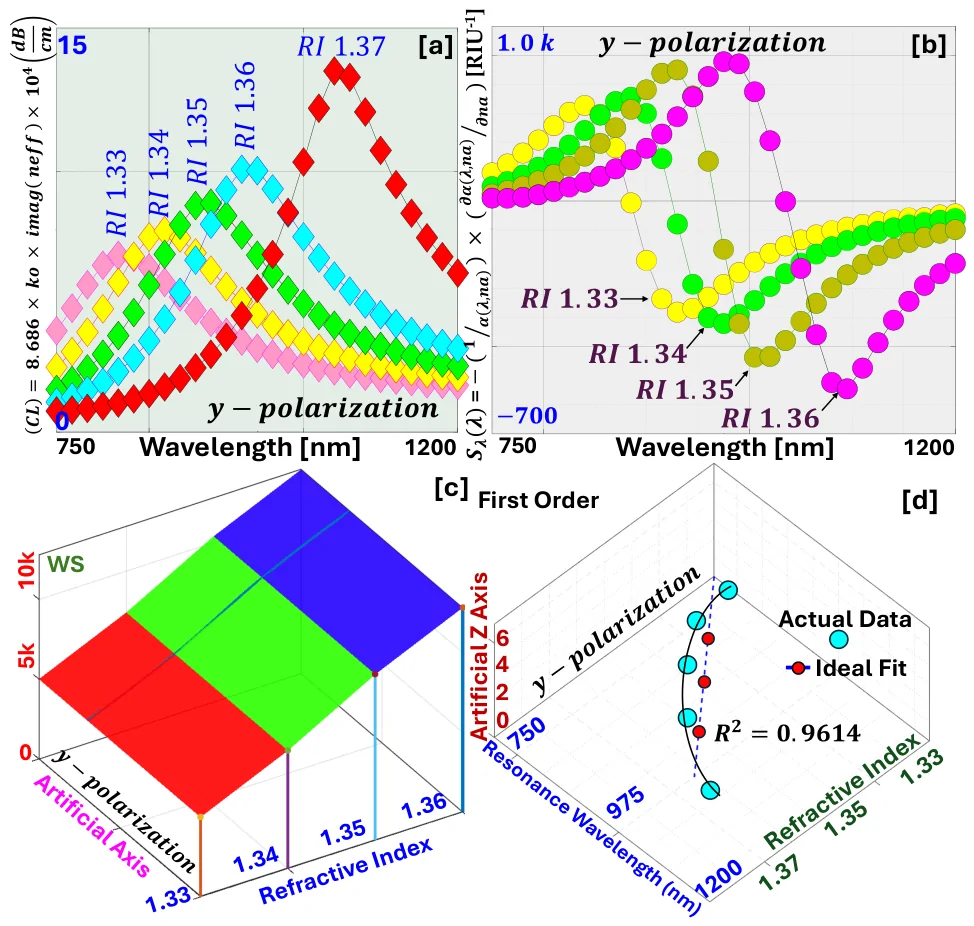
Sensing behavior of the sensor model for y-pol. (**a**) CL; (**b**) AS; (**c**) WS; (**d**) relationship between RW and RI corresponding to y-pol.

Similarly, Figure 11a represents the CL of 8.2412, 9.0605, 9.9879, 11.213 and 14.5579 dB/cm for water, urine sample-normal, T-47D breast cancer, MM6- leukemia and DU-145 prostate cancer, respectively, corresponding to y-pol. at RW of 870, 914.43, 952.75, 989.77, and 1080.46 nm, respectively. Figure 11b represents AS of 381.737, 421.089, 534.213, and $645.087\;{RIU}^{-1}$ for water, urine sample-normal, T-47D breast cancer, and MM6- leukemia, respectively, corresponding to y-pol. Figure 11c represents the WS of 4443, 3832, 3702, and 9069 nm/RIU for water, urine sample-normal, T-47D breast cancer, and MM6- leukemia, respectively, corresponding to y-pol. Figure 11d represents the first-order linear relationship between the RW and RI. The linear relationship is expressed by Equation (10).

Where $p_{1}=4963$ and $p_{2}=-5738$. The goodness of fit can be expressed by parameters like SSE=987.7, RMSE=18.14, adjusted $R^{2}=0.9486$ and $R^{2}\;=\;0.9614$ showcasing good fitting between the sensor parameters corresponding to y-pol. The SR of $2.2507\times 10^{-5}$, $2.6096\times 10^{-5}$, $2.7012\times 10^{-5}$ and $1.1027\times 10^{-5}$ is obtained for the water, urine sample, T47- breast cancer, and MM6- leukemia for y-pol. respectively. Finally, the FWHM of 189.31, 183.43, 179.0 and 171.08 nm is obtained resulting FOM of 23.41, 20.89, 20.68 and $53.01\;{RIU}^{-1}$ for water, urine sample, T-47D breast cancer and MM6 leukemia, respectively, corresponding to y-pol.

Table 4 summarizes the calculated sensing parameters of the proposed biosensor for y-pol. conditions.

**Figure 12:**
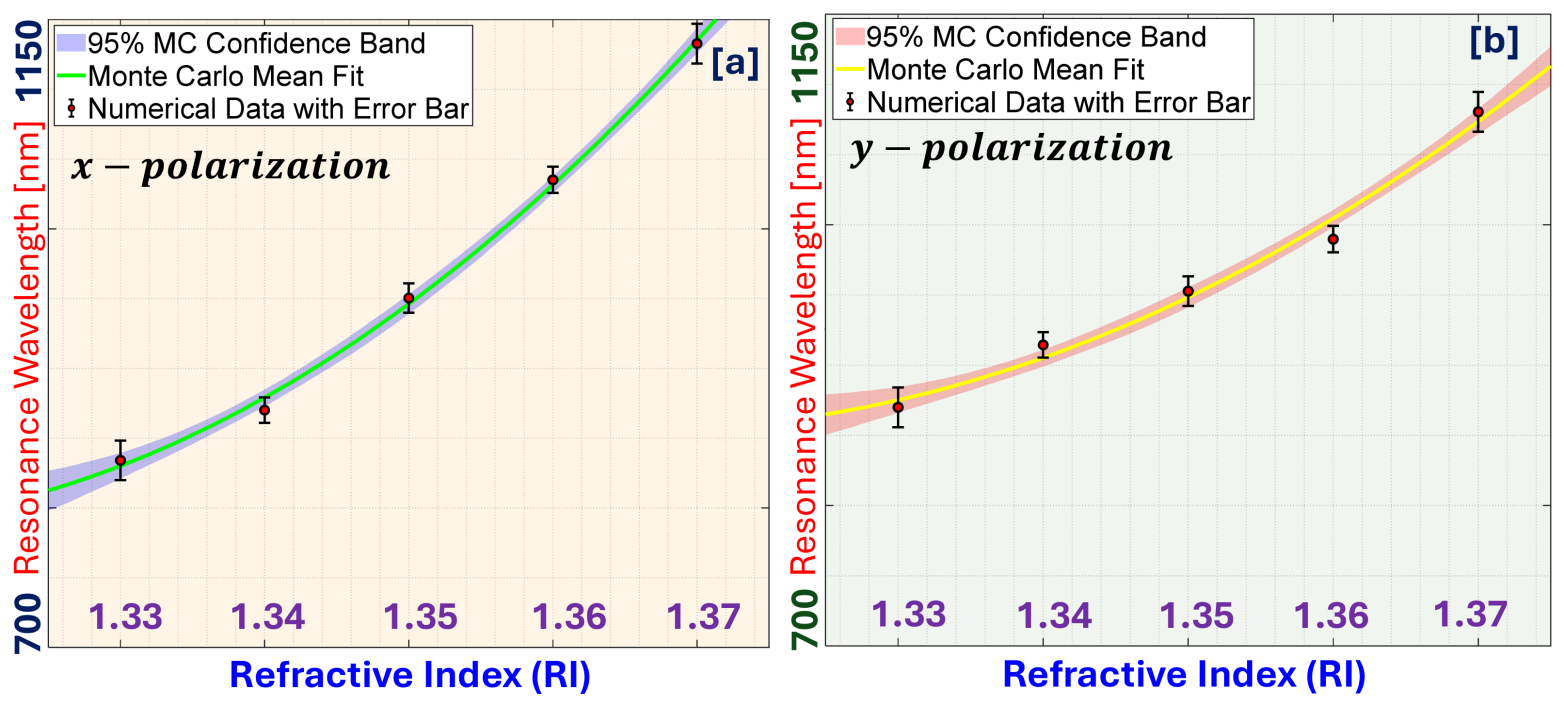
Monte Carlo error-bar and confidence-band analysis of the RW versus analyte RI relationship for (**a**) x-polarization and (**b**) y-polarization. The mean fitted curve, 95% confidence band, and uncertainty error bars obtained from 2000 realizations are presented to assess the robustness of the extracted sensitivity.

To further evaluate the reliability of the RW−RI relationship, a Monte Carlo-based uncertainty analysis comprising 2000 realizations is performed for both polarization modes. The resulting 95% confidence bands and error bars are presented in Figure 12a,b corresponding to x-pol. and y-pol., respectively. The relatively narrow confidence intervals indicate good robustness of the fitted calibration models and support the reliability of the extracted sensitivity values.

## 7. Effect of Increased Material Thickness on Sensor Performance

### 7.1. Evaluation of Sensing Performance at Increased Thickness of Plasmonic Material

This section showcases the result of the sensing performance of the proposed sensor when the thickness of the plasmonic materials is increased beyond the optimum thickness. The thickness of the plasmonic materials is increased by 5 nm, due to which the new thickness of Au and $TiO_{2}$ is increased to become 50 nm and 90 nm respectively.

**Figure 13:**
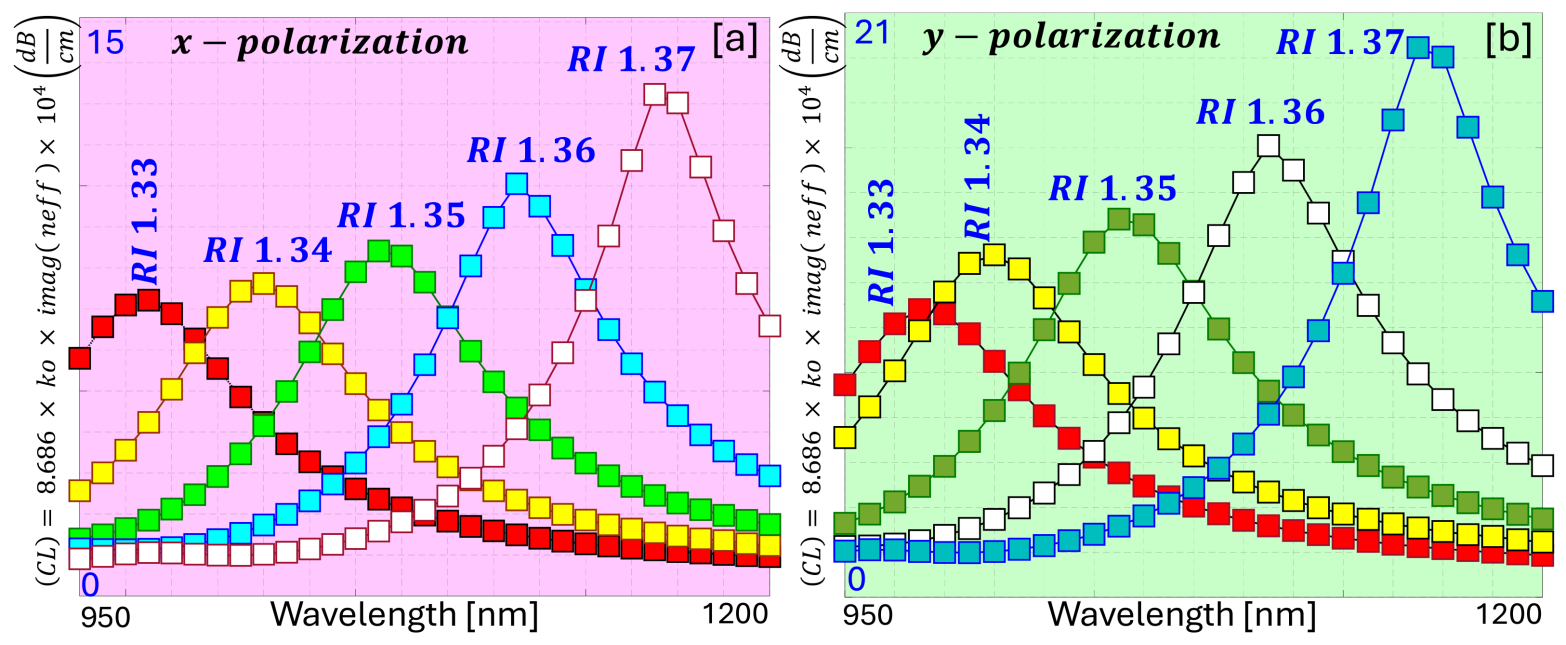
CL of the sensor model with increased thickness of plasmonic materials: (**a**) x-pol and (**b**) y-pol.

Figure 13a represents the CL of 7.6326, 8.5637, 9.6891, 11.1321, and 12.92 dB/cm for water, urine sample-normal, T-47D breast cancer, MM6- leukemia and DU145 prostate cancer, respectively, corresponding to x-pol. Similarly, Figure 13b represents the CL of 12.3262, 13.8061, 15.6219, 17.9055, and 20.8131 dB/cm for water, urine sample-normal, T-47D breast cancer, MM6- leukemia and DU145 prostate cancer, respectively, corresponding to y-pol.

**Figure 14:**
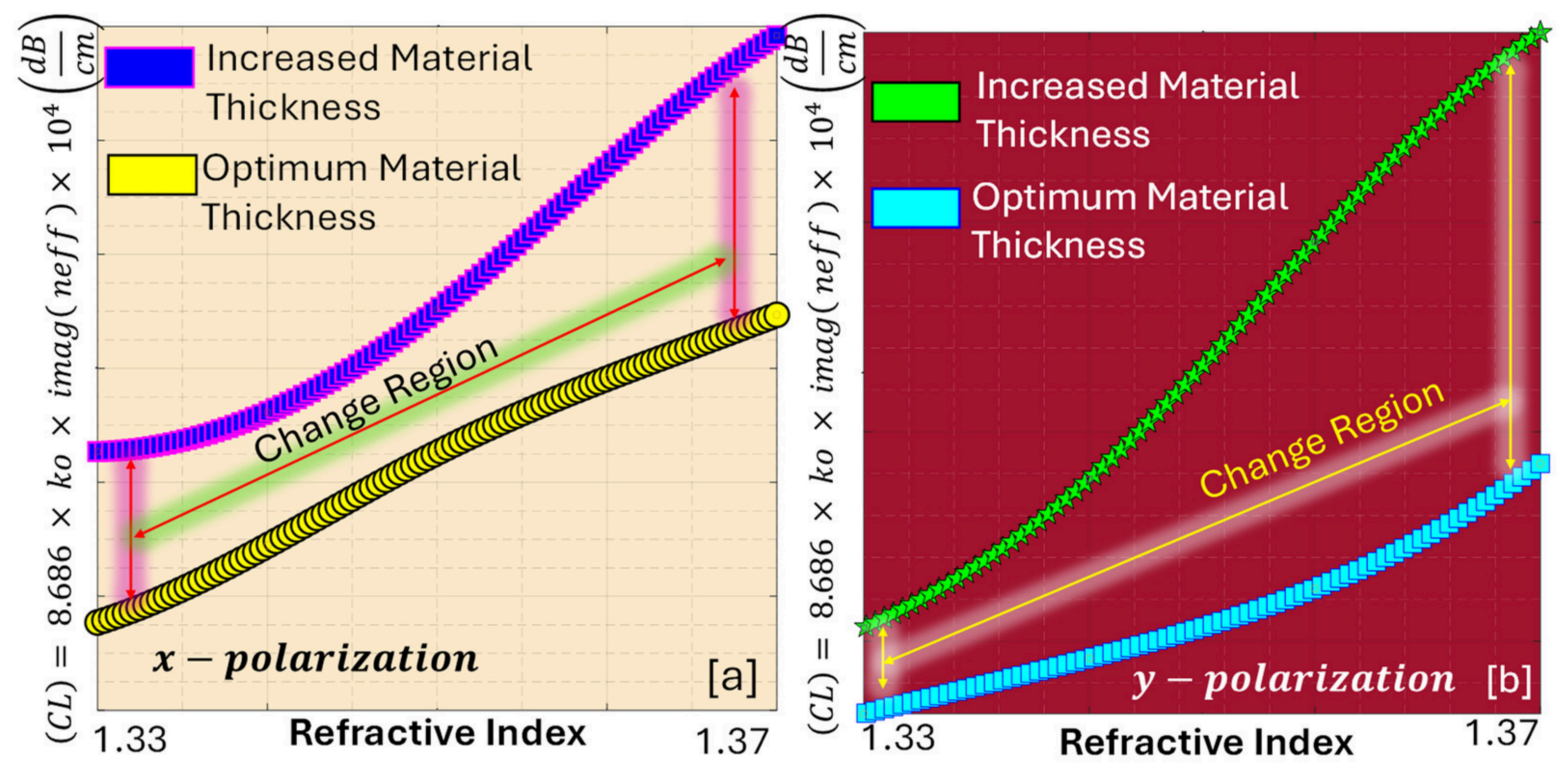
Comparison of CL for the sensor model with optimum and increased thickness of plasmonic materials: (**a**) x-pol and (**b**) y-pol.

Figure 14a,b represents the behavior of the CL at the optimized thickness and at the improved thickness of plasmonic materials for x-pol. and y-pol., respectively. Here, it can be observed clearly that when the thickness of plasmonic materials is increased beyond the optimum thickness, an increase in the CL for the sensor model is observed for x-pol. and y-pol. , respectively.

**Figure 15:**
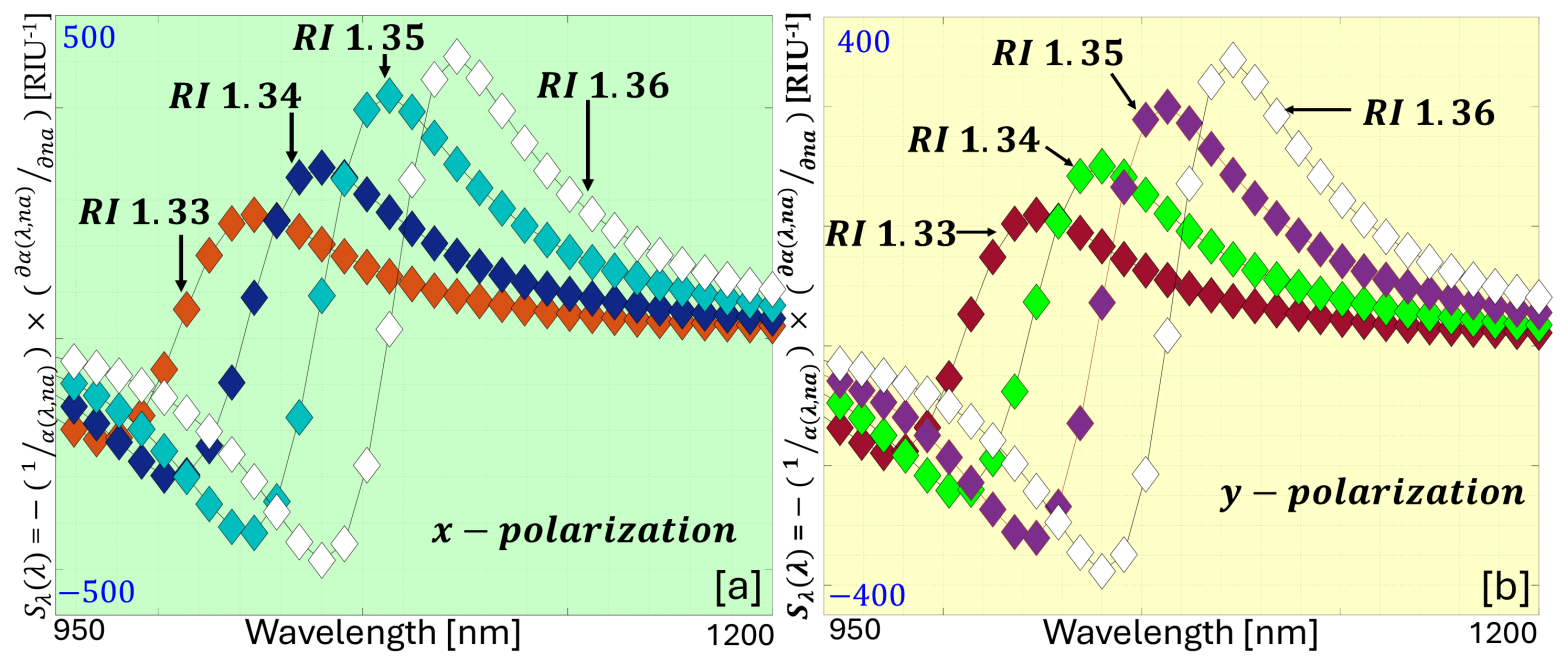
AS of the sensor model with increased thickness of plasmonic materials: (**a**) x-pol and (**b**) y-pol.

Figure 15 represents the AS corresponding to increased thickness of plasmonic materials for x-pol. and y-pol. respectively. Figure 15a represent AS of 225.031, 300.143, 421.624, and $480.913\;{RIU\;}^{-1}$ for water, urine sample-normal, T-47D breast cancer, and MM6- leukemia, respectively, corresponding to x-pol. Figure 15b represents AS of 283.283, 320.169, 341.06, and $376.898\;{RIU}^{-1}$ for water, urine sample-normal, T-47D breast cancer, and MM6- leukemia, respectively, corresponding to y-pol.

**Figure 16:**
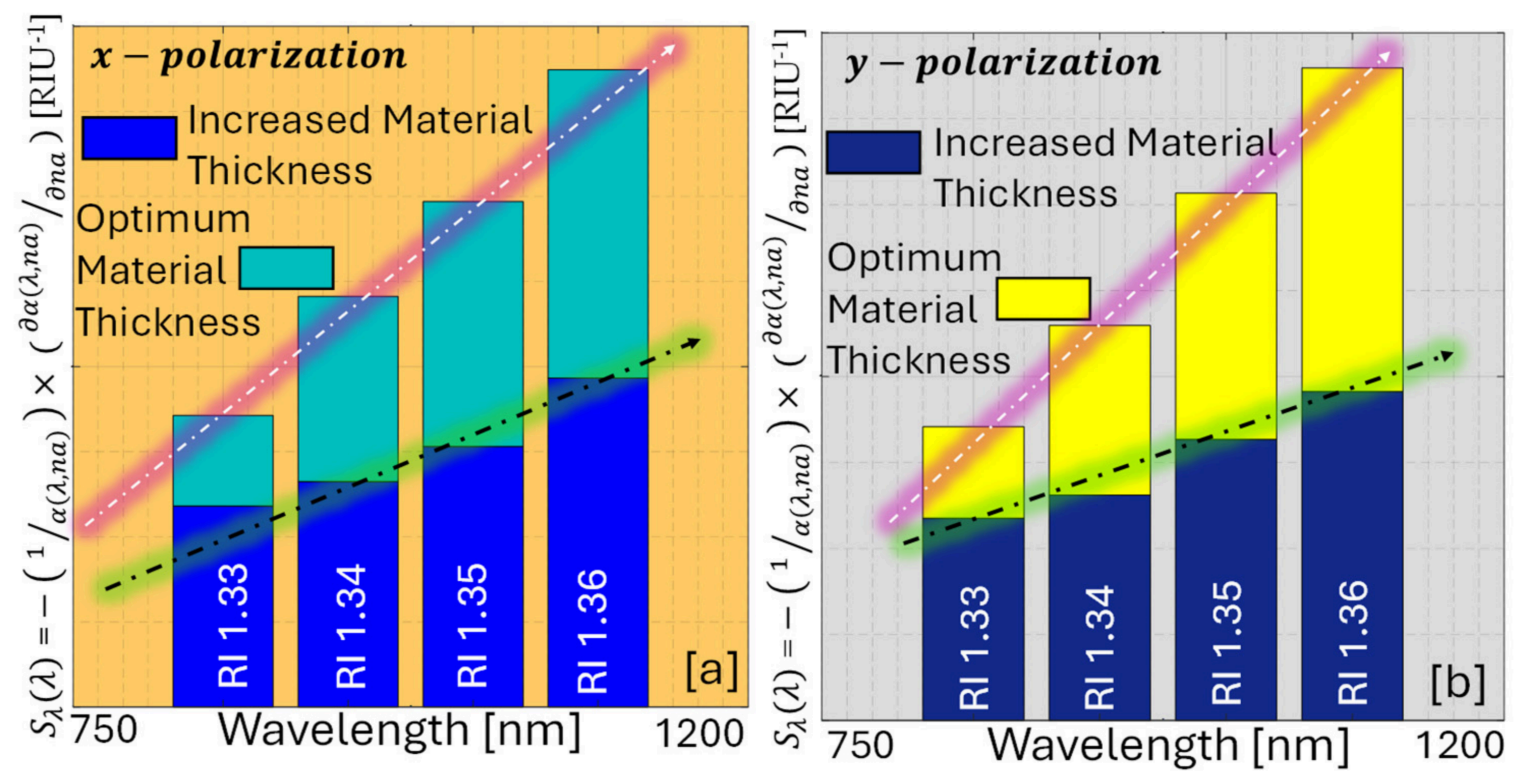
Comparison of the AS of the sensor model with optimum and increased thickness of plasmonic materials: (**a**) x-pol and (**b**) y-pol.

Figure 16a,b represents the behavior of the AS at optimized thickness and at improved thickness of plasmonic materials for x-pol. and y-pol. respectively. Here, it can be observed clearly that when the thickness of plasmonic materials is increased beyond optimum thickness, a decrease in the AS for the sensor model is observed.

Thus, it can be observed that when the thickness of the plasmonic material exceeds its optimum value, the penetration of the evanescent field into the plasmonic layer is reduced. Due to this, the coupling and phase matching between the core-guided mode and the SPP mode gets weakened. Consequently, the CL characteristics deteriorate, leading to a reduction in WS and AS of the proposed sensor and other sensing parameters.

### 7.2. Fabrication Tolerance Assessment of the Proposed Sensor

This section of the article presents results of the fabrication tolerance assessment (FTA) for the proposed sensor. FTA is an essential aspect in assessing the practical feasibility of any sensor model. In the early stages of PCF–SPR sensor development, a fabrication tolerance of ±1% was also considered acceptable due to limited resources and fabrication challenges. However, with recent advancements in micro- and nano-fabrication technologies, researchers have successfully achieved tolerance levels as high as ±10% without significantly compromising sensor performance. For the FTA analysis, the key geometrical parameters such as air hole diameter and Λ are systematically varied within a tolerance range of approximately ±1% to ±10%. This variation helps to evaluate the robustness of the sensor against fabrication imperfections and ensures reliable real-world implementation. Thus, in this section of the article, results of variations in CL and AS by varying the geometrical dimension of the air holes and Λ are presented. The proposed sensor consists of two dimensions of air holes, $d_{1}$ and $d_{2}$. Now, the dimensions of both the air holes are varied by ±10%, and changes in the sensor sensing parameters like CL and AS are analyzed.

**Figure 17:**
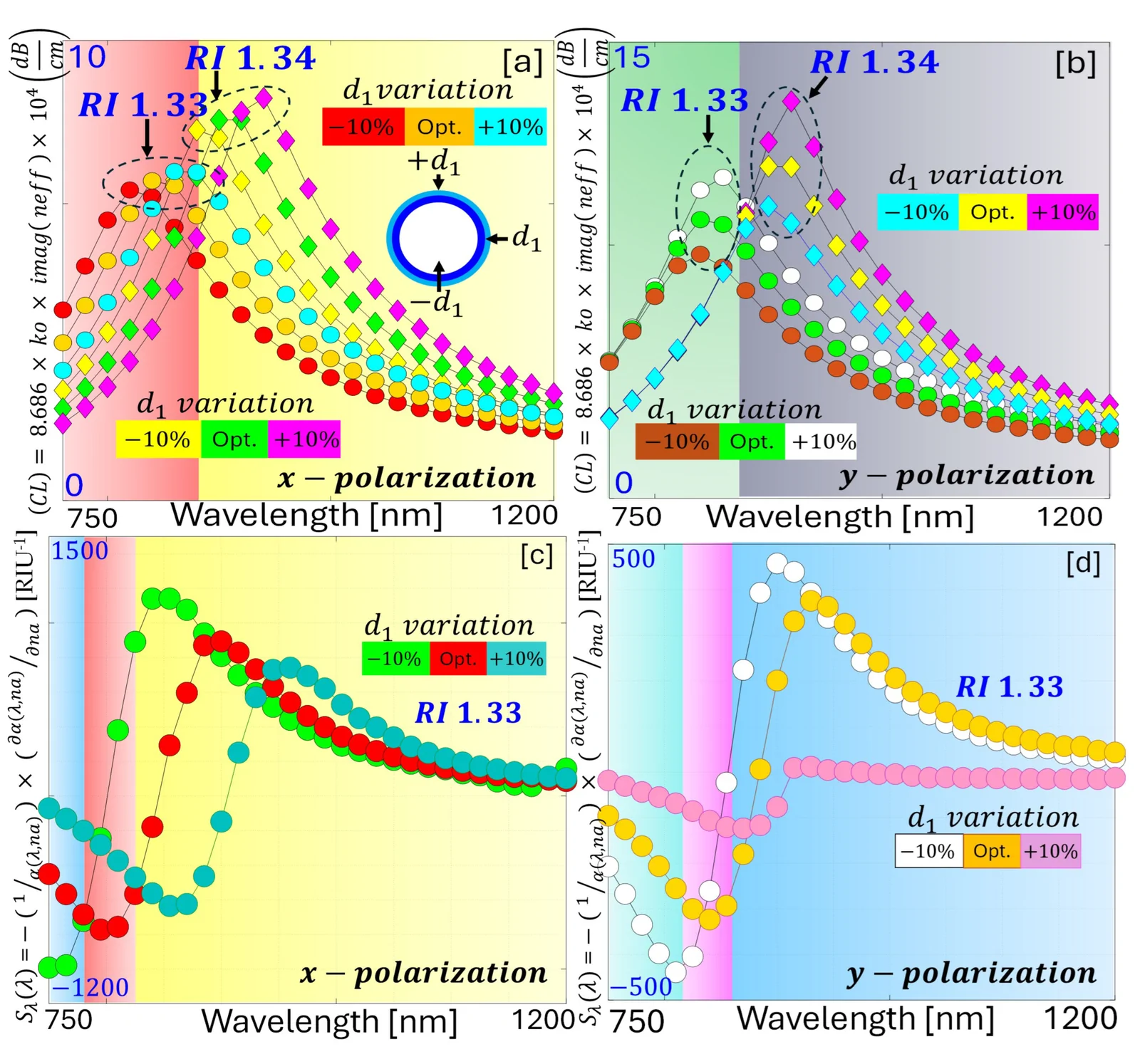
FTA analysis by varying air hole diameter (d_1_) by ±10%. (**a**) CL variation for x-pol. (**b**) CL variation for y-pol. (**c**) AS variation for x-pol. (**d**) AS variation for y-pol.

Firstly, the dimensions of the air holes $d_{1}$ are varied by ±10%  then the new dimensions become 1.65 µm and 1.35 µm, respectively. So, for the x-pol., an increase in CL of 0.0523 dB/cm and a decrease in CL of 0.3389 dB/cm is observed corresponding to RI 1.33, against optimum CL of 4.1053 dB/cm. Similarly, for the RI 1.34  an increase in CL of 0.6313 dB/cm and a decrease in CL of 0.1763 dB/cm is observed, against optimum CL of 4.5303 dB/cm respectively. This change in CL is represented by Figure 17a. Now, corresponding to y-pol. for the RI 1.33 an increase in CL of 0.0131 dB/cm and a decrease in CL of 0.5599 dB/cm is observed, against optimum CL of 8.2413 dB/cm. Similarly, for the RI 1.34, an increase in CL of 0.0378 dB/cm and a decrease in CL of 0.3048 dB/cm is observed, against optimum CL of 9.0605 dB/cm. This change in CL is represented by Figure 17b. Figure 17c presents the behavior of AS of the proposed sensor by varying the dimension of the air holes; here it can be observed that AS has decline by $29.194\;{RIU}^{-1}$ and increased by $185.029\;{RIU}^{-1}$ by changing the air hole dimension by $-10\%\;d_{1}$ and $+10\%\;d_{1}$ respectively, corresponding to x-pol. for RI 1.33. Finally, in Figure 17d, it can be observed that AS has decline by $265.311\;{RIU}^{-1}$ and increased by $57.255\;{RIU}^{-1}$, respectively, by changing the air hole dimension by $-10\%\;d_{1}$ and $+10\%\;d_{1}$ respectively, corresponding to y-pol. for RI 1.33.

**Figure 18:**
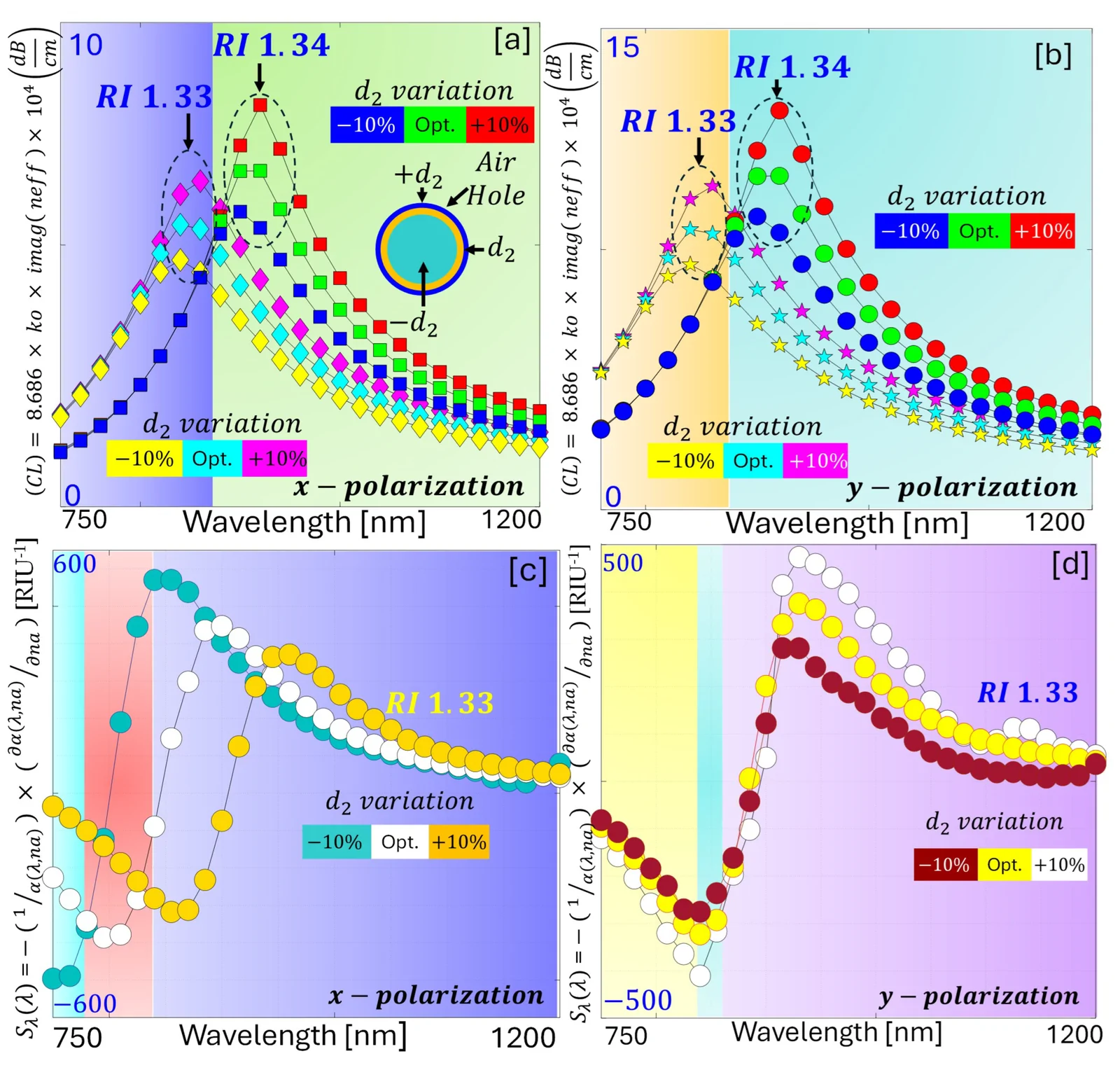
FTA analysis by varying air hole diameter (d_2_) by ±10%. (**a**) CL variation for x-pol. (**b**) CL variation for y-pol. (**c**) AS for x-pol. (**d**) AS for y-pol.

Secondly, the dimension of air holes $d_{2}$ is varied by ±10% and then the new dimensions become 1.32 µm and 1.08 µm respectively. So, for the RI 1.33, an increase in CL of 0.421 dB/cm and a decrease in CL of 0.4119 dB/cm is observed corresponding to x-pol. against optimum CL of 4.1053 dB/cm. Similarly, for the RI 1.34, an increase in CL of 0.4624 dB/cm and a decrease in CL of 0.541 dB/cm is observed, against optimum CL of 4.5303 dB/cm corresponding to x-pol. respectively. This change in CL is represented by Figure 18a. Now, corresponding to y-pol. for the RI 1.33 an increase in CL of 0.8184 dB/cm and a decrease in CL of 0.8248 dB/cm is observed, against optimum CL of 8.2413 dB/cm. Similarly, for the RI 1.34, an increase in CL of 0.9048 dB/cm and a decrease in CL of 0.911 dB/cm is observed, against optimum CL of 9.0605 dB/cm. This change in CL is represented by Figure 18b. Figure 18c presents the behavior of the AS of the proposed sensor by varying the dimensions of the air holes; here it can be observed that AS has decline by $13.812\;{RIU}^{-1}$ and increased by $185.029\;{RIU}^{-1}$ by changing the air hole dimension by $-10\%\;d_{2}$ and $+10\%\;d_{2}$ respectively, corresponding to x-pol. for RI 1.33. Finally, in Figure 18d, it can be observed that AS has decline by $106.376\;{RIU}^{-1}$ and increased by $29.028\;{RIU}^{-1}$, respectively, by changing the air hole dimension by $-10\%\;d_{2}$ and $+10\%\;d_{2},$ respectively, corresponding to y-pol. for RI 1.33.

Finally, the Λ is varied by ±10%, then the new dimensions of Λ becomes 2.475 µm and 2.025 µm for +10% Λ and −10% Λ, respectively.

**Figure 19:**
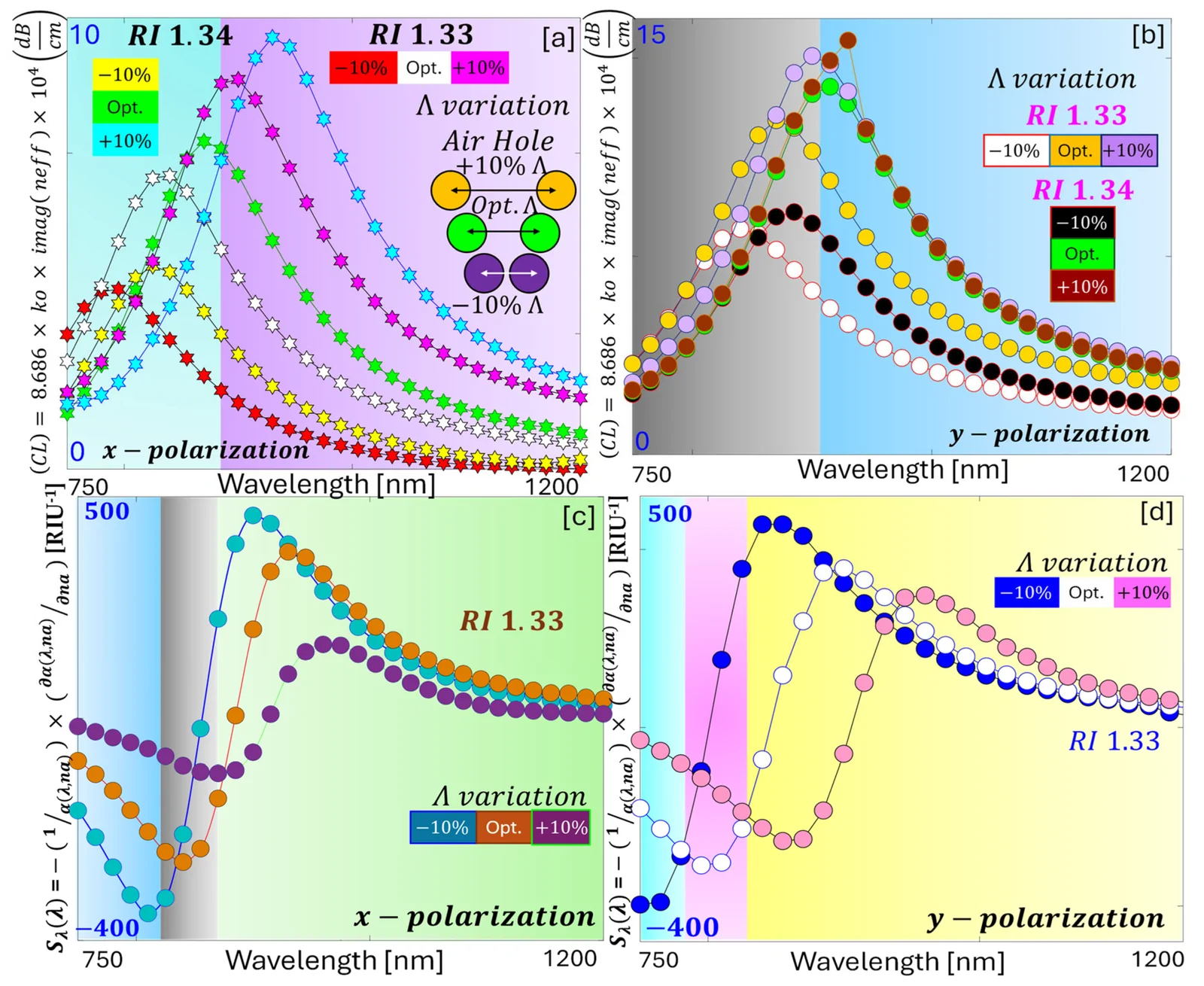
FTA analysis by varying Λ by ±10%. (**a**) CL variation for x-pol. (**b**) CL variation for y-pol. (**c**) AS for x-pol. (**d**) AS for y-pol.

So, for x-pol., an increase in CL of 1.7118 dB/cm and decrease in CL of 0.6133 dB/cm is observed for +10% Λ and −10% Λ corresponding to RI 1.33, against optimum CL of 4.1053 dB/cm. Similarly, for the RI 1.34, an increase in CL of 1.7803 dB/cm and decrease in CL of 0.7538 dB/cm is observed, for +10% Λ and −10% Λ corresponding to optimum CL of 4.5303dB/cm, respectively. This change in CL is represented by Figure 19a.

Considering y-pol. for the RI 1.33 an increase in CL of 1.8139 dB/cm and a decrease in CL of 2.5470 dB/cm is observed for +10% Λ and −10% Λ corresponding to optimum CL of 8.2413 dB/cm. Similarly, for the RI 1.34 an increase in CL of 1.3858 dB/cm and a decrease in CL of 2.9415 dB/cm is observed for +10% Λ and −10% Λ corresponding to optimum CL of 9.0605 dB/cm. This change in CL is represented by Figure 19b.

Figure 19c presents the behavior of the AS of the proposed sensor by varying the dimension of Λ, by ±10% here it can be observed that AS has decline by $180.473\;{RIU}^{-1}$ and increased by $135.151\;{RIU}^{-1}$ by changing Λ dimension by −10% Λ and +10% Λ respectively, corresponding to x-pol. for RI 1.33.

Finally, Figure 19d presents the behaviour of the AS of the proposed sensor by varying the dimension of Λ here it can be observed that AS decline by $64.091\;{RIU}^{-1}$ and increased by $107.126\;{RIU}^{-1}$ respectively by changing the Λ by −10% Λ and +10% Λ, corresponding to y-pol. for RI 1.33.

Thus, it can be observed that even for a small change in the geometrical parameters, the sensing parameters show minor to major changes. The present FTA analysis focuses on the key geometrical parameters that are expected to exert the strongest influence on plasmonic coupling and sensing performance. A more comprehensive tolerance study can be performed involving additional parameters such as variation in $TiO_{2}$ thickness, and analyte-channel dimensions may be considered in future work. Other sensing parameters that can be used in evaluating the overall sensing performance and sensitivity of the proposed sensor model include the effective sensor length and birefringence behavior, which basically influence the polarization-dependent responses and enhance the detection accuracy. Therefore, the proposed sensor demonstrates strong resilience to structural variations while maintaining high sensitivity to RI changes in the cancerous cell samples. This indicates that the proposed biosensor, when fabricated, carries significant potential to be used for practical biomedical applications, particularly in early-stage cancer detection and diagnostic sensing systems. Finally, Table 5 presents a comparison of the sensing parameters of the proposed sensor with previously reported cancer biosensors of recent times.

There are some important considerations to be noted while performing SPR sensing via the proposed PCF. The detection capability of the proposed biosensor is primarily governed by its calculated SR and wavelength resolution of the OSA, rather than the RI interval selected for the simulations. In this study, an RI step of 0.01 RIU was chosen to evaluate the sensing performance over the RI range relevant to the investigated cancer cell samples. Future studies can consider smaller RI increments to provide a more detailed analysis of the sensor response for cancer detection applications. A second important consideration is that, like most RI-based biosensors, the proposed sensor may also be affected by non-specific adsorption and biofouling when operating in complex biological environments. Thus, in real-time sensing applications, these effects can be minimized through appropriate surface functionalization, anti-fouling coatings, selective biorecognition layers, and through sample pretreatment procedures. Future experimental studies should therefore focus on integrating these strategies to improve the selectivity and reliability of the proposed biosensor. Finally, it is important to note that RI alone may not always serve as a disease-specific biomarker, as similar RI variations can arise from different physiological or pathological conditions. Therefore, in practical clinical applications, the sensing response of the proposed biosensor may be complemented with orthogonal biomarkers, such as specific proteins, nucleic acids, circulating tumor markers, or cellular characteristics, to improve diagnostic specificity. In addition, emerging machine-learning and artificial-intelligence-based classification approaches can integrate multiple sensing features, including RW, CL, AS, and WS, to enhance disease discrimination and support more reliable clinical decision-making. The incorporation of such strategies represents a promising direction for future translational and experimental studies.

## 8. Conclusions

This study presents a dual-polarized tetra-core PCF–SPR sensor designed for analyzing water, urine sample-normal, T-47 D breast cancer, MM6- leukemia and DU145 prostate cancer based on their RI variations. The proposed sensor can effectively detect the variations in the cancer cells by achieving high sensitivity and performance across different sensing parameters. The sensor model has obtained a lowest CL varying up to 4.1053 dB/cm for x-pol., and 8.2412 dB/cm for y-pol., respectively. A high WS of 9769 and 9069 nm/RIU is obtained for x-pol. and y-pol. respectively. An AS of 623.182 and $645.087\;{RIU}^{-1}$ is obtained for x-pol. and y-pol., respectively. SR in the order of ${10\;}^{-5}$ is also obtained for both polarization modes and linear fitting; between the RW and RI has obtained $R^{2}$ of 0.97 and 0.96 r x-pol. and y-pol., respectively and finally, FOM of 60.17 and $53.01\;{RIU}^{-1}$ is obtained for x-pol. and y-pol. respectively. Thus, the proposed PCF–SPR biosensor exhibits a dynamic range of 0.04 RIU. Beside real-time analysis of the cancer cells is performed based on the RI values for which the proposed sensor is designed. Thus, it can be observed that the proposed sensor, with its tetra-core structure, dual-polarization capability, and wide-ranging sensing parameters, presents a good approach for analyzing cancer cells based on RI values for healthcare monitoring.

## Figures and Tables

**Table 1:** Literature-reported RI values, representative biological samples, and their diagnostic relevance in cancer biosensing.

RI	Sample	Biological Significance	Diagnostic Relevance	Ref.
1.33	Water	Calibration and baseline medium	Sensor baseline/reference	[43,44]
1.34	Normal Urine	Healthy physiological condition	Healthy control sample	[43,44]
1.35	Blood Plasma	Normal plasma protein concentration	Plasma-based cancer biomarker studies (Breast, lung, colorectal)	[43,44]
1.36	WBC rich sample	Elevated cellular concentration	Leukemia and immune cell-related diagnostics	[43,44]
1.37	EV/Exosome rich urine	Elevated extracellular vesicle concentration	Urinary EV-based cancer diagnostics (Prostate, bladder, kidney)	[43,44]

**Table 2:** Relationship between cancer types and related biomarkers.

Cancer Type	Body Fluid Sample	Related Biomarker	Ref.
Bladder cancer	Urine	Urinary EVs, exosomes	[45,46]
Prostate cancer	Urine, plasma	PSA-associated EVs	[47,48]
Kidney cancer	Urine	Tumor-derived vesicles	[49,50]
Breast cancer	Blood plasma	Circulating exosomes	[51,52]
Lung cancer	Blood plasma	Tumor-derived exosomes	[53,54]
Colorectal cancer	Blood plasma	Extracellular vesicles	[55,56]

**Table 3:** Summary of the sensing parameters for x-polarization.

RI	∆ RI	RW (nm)	Peak CL (dB/cm)	WS (nm/RIU)	AS (RIU^−1^)	SR (RIU)	FWHM (nm)	FOM (RIU^−1^)
1.33	0.01	834.06	4.1053	3603	303.834	2.7755 × 10^−5^	166.04	21.69
1.34	0.01	870.09	4.5303	8039	471.384	1.12439 × 10^−5^	187.62	42.84
1.35	0.01	950.48	5.4944	8467	550.377	1.1811 × 10^−5^	183.79	46.06
1.36	0.01	1035.15	7.1458	9796	623.182	1.0236 × 10^−5^	162.34	60.17
1.37	0.01	1132.84	9.2657	NA	NA	NA	NA	NA

**Table 4:** Summary of the sensing parameters for y-polarization.

RI	∆ RI	RW (nm)	Peak CL (dB/cm)	WS (nm/RIU)	AS (RIU^−1^)	SR (RIU)	FWHM (nm)	FOM (RIU^−1^)
1.33	0.01	870.0	8.2412	4443	381.737	2.2507 × 10^−5^	189.31	23.41
1.34	0.01	914.43	9.0605	3832	421.089	2.6096 × 10^−5^	183.43	20.89
1.35	0.01	952.75	9.9879	3702	534.213	2.7012 × 10^−5^	179.0	20.68
1.36	0.01	989.77	11.213	9069	645.087	1.1027 × 10^−5^	171.08	53.01
1.37	0.01	1080.46	14.5579	NA	NA	NA	NA	NA

**Table 5:** Sensing parameters comparison of the proposed sensor with previously reported sensors.

Ref	Shape	RI	Pol.	WS (nm/RIU)	AS (RIU^−1^)	SR (RIU)	FOM (RIU^−1^)	DR	R^2^/ Order	Biological Analysis
[76]	Grooved	1.36–1.399	x-pol.	2142.86	632.50	10^−5^	26.78	0.039	0.904/ I order	NO
[77]	EMD	1.38–1.401	x-pol.	13,257.20	---	---	36.52	0.021	---	NO
[78]	EMD	1.36–1.401	x-pol. y-pol.	5714.28	899.248	10^−5^	---	0.041	---	NO
[79]	Grooved	1.368–1.40	y-pol.	2142.86	1058.039	4.6 × 10^−5^	---	0.032	---	NO
[80]	D shape	1.34	y-pol.	10,000	235,882	---	---	---	---	NO
[81]	EMD	1.368–1.40	y-pol.	5714.29	599.53	4.0 × 10^−5^	---	0.032	---	NO
[82]	Grooved	1.37–1.41	x-pol. y-pol.	7800 11,700	--- ---	--- ---	---	0.040	--- ---	NO
Proposed	EMD	1.33–1.37	x-pol. y-pol.	9769 9069	623.182 645.087	1.02 × 10^−5^ 1.10 × 10^−5^	60.17 53.01	0.040	0.97/I order 0.96/I order	Yes

## Data Availability

The raw data supporting the conclusions of this article will be made available by the authors on request.
